# The effect of medical explanations from large language models on diagnostic accuracy in radiology

**DOI:** 10.1038/s41746-026-02619-0

**Published:** 2026-04-23

**Authors:** Philipp Spitzer, Daniel Hendriks, Jan Rudolph, Sarah Schlaeger, Jens Ricke, Niklas Kühl, Boj Friedrich Hoppe, Stefan Feuerriegel

**Affiliations:** 1 Karlsruhe Institute of Technology, Karlsruhe, Germany; 2 Department of Radiology, LMU University Hospital, LMU Munich, Munich, Germany; 3 University of Bayreuth, Bayreuth, Germany; 4 Fraunhofer FIT, Bayreuth, Germany; 5 Munich Center for Machine Learning, Munich, Germany; 6 LMU Munich, Munich, Germany

**Keywords:** Computational biology and bioinformatics, Diseases, Health care, Mathematics and computing, Medical research

## Abstract

Large language models (LLMs) are increasingly used by physicians for diagnostic support. A key advantage of LLMs is the ability to generate explanations that can help physicians understand the reasoning behind a diagnosis. However, the best-suited format for LLM-generated explanations remains unclear. In this large-scale study, we examined the effect of different formats for LLM explanations on clinical decision-making. For this, we conducted a randomized experiment with radiologists reviewing patient cases with radiological images (*N* = 2020 assessments). Participants received either no LLM support (control group) or were supported by one of three LLM-generated explanations: (1) a *standard output* providing the diagnosis without explanation; (2) a *differential diagnosis* comparing multiple possible diagnoses; or (3) a *chain-of-thought* explanation offering a detailed reasoning process for the diagnosis. We find that the format of explanations significantly influences diagnostic accuracy. The chain-of-thought explanations yielded the best performance, improving the diagnostic accuracy by 12.2% compared to the control condition without LLM support (*P* = 0.001). The chain-of-thought explanations are also superior to the standard output without explanation ( + 7.2%; *P* = 0.040) and the differential diagnosis format ( + 9.7%; *P* = 0.004). We further assessed the robustness of these findings across case difficulty and different physician backgrounds, such as general vs. specialized radiologists. Evidently, in the controlled setting of our vignette study, explaining the reasoning for a diagnosis helps physicians to identify and correct potential errors in LLM predictions and thus improve overall decisions. Altogether, the results highlight the importance of explanations in medical LLMs to support the reasoning processes of physicians, so that medical LLMs can improve diagnostic performance and, ultimately, patient outcomes.

## Introduction

Large language models (LLMs), a form of artificial intelligence (AI) capable of producing human-like text, are becoming increasingly common in clinical workflows^1,2^, including tasks such as assisting with diagnosis^3–7^. A particular promise of LLMs is to improve patient outcomes by reducing diagnostic errors^8^, a major source of preventable harm^9–11^, but diagnostic errors still remain a major concern: according to one estimate, over half a million people die or suffer permanent disability each year as a result in the US^9^.

Existing research has extensively evaluated the diagnostic accuracy of LLMs in clinical practice, often finding that LLMs perform as well as human physicians and, in some cases, achieve superhuman performance^5,6,12–14^. Other studies have examined the diagnostic accuracy of physicians when supported by LLMs compared to when they work without LLM assistance^4,7,12,15^. However, these studies primarily assess the correctness of LLM-generated diagnoses but neglect how explanations provided by the LLMs influence the decision-making of physicians. Notwithstanding, a key strength of LLMs is that they can explain in natural language why a certain diagnosis seems likely. Such explanations can help physicians understand and verify the rationale behind a suggested diagnosis. Yet, the impact of different explanation formats on medical decision-making is unclear. Thus, we analyze how different formats for LLM-generated explanations influence the diagnostic accuracy of physicians.

We hypothesize that the format of LLM-generated explanations affects the diagnostic accuracy of physicians. On the one hand, explanations may improve diagnostic accuracy by helping physicians understand the reasoning behind a diagnosis. For instance, explanations with a differential diagnosis align with standard clinical practice, where differential diagnoses help reduce cognitive biases such as premature closure and anchoring by encouraging consideration of alternative diagnoses^16,17^. Similarly, LLMs can generate explanations through chain-of-thought reasoning^18^, where the LLM is asked to provide a step-by-step explanation so that a physician can verify its plausibility against their domain knowledge. Chain-of-thought explanations were originally introduced as a prompting strategy that encourages large language models to present intermediate reasoning steps leading to a final answer. Prior work has shown that such step-by-step explanations can improve performance on complex reasoning tasks and increase transparency of model outputs, making them easier for humans to inspect and evaluate^18,19^. Subsequent studies have demonstrated that variations of chain-of-thought prompting can further enhance reasoning consistency and robustness across tasks^20^. On the other hand, explanations can also mislead physicians^21–23^. A key risk is automation bias, where humans overly trust machine-generated outputs such as LLM-generated advice^24,25^. This risk is particularly pronounced for LLM-generated explanations, as LLMs tend to produce explanations that sound convincing but contain errors, a phenomenon known as “hallucinations”^26,27^. Consequently, explanations, especially if detailed but incorrect, may also lead to diagnostic errors. Overall, LLM-generated explanations may support or hinder medical decision-making. However, the best-suited design of LLM-generated explanations is unclear.

**Fig. 1:**
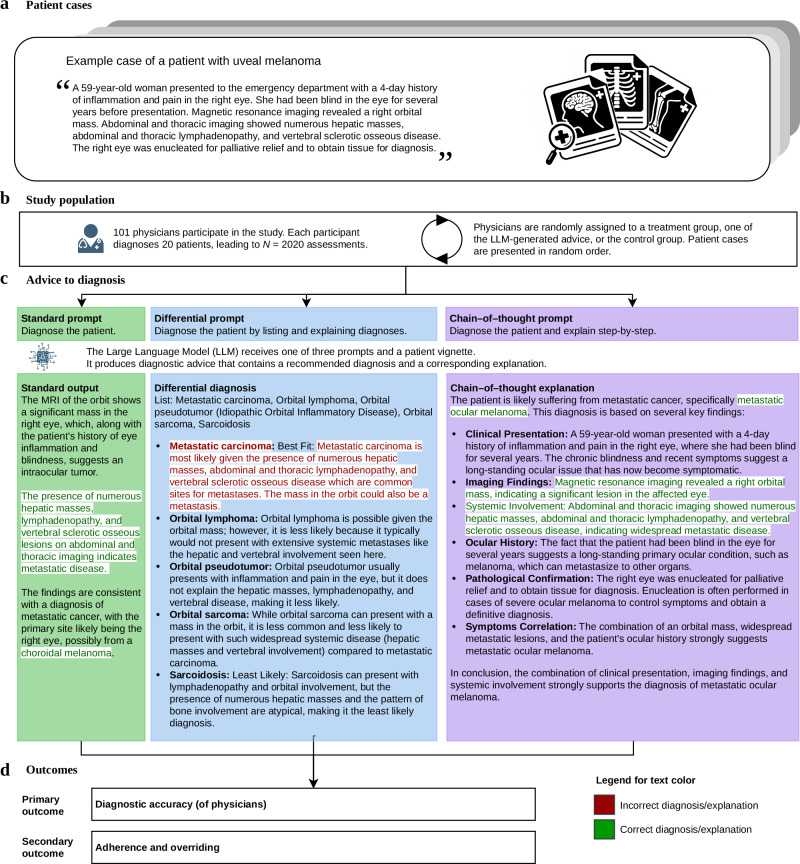
Research design. **a** We selected 20 real-world patient cases from the *New England Journal of Medicine Image Challenge*^72^. Each patient case includes a textual description and at least one radiological image (i.e., computed tomography [CT] or magnetic resonance imaging [MRI]). To ensure our evaluation was not compromised by data leakage, we conducted a memorization test^33^ in which GPT-4 was prompted to continue truncated patient descriptions. This yielded consistently low similarity scores, indicating that the model had not memorized our patient cases. **b** We recruited 101 radiologists from the U.S. who were asked to provide diagnoses for each case and who were randomly assigned to either a control group (no LLM support) or one of three treatment groups (with LLM support). **c** We designed prompts to produce LLM-based explanations with different formats (i.e., *standard output*, *differential diagnosis*, and *chain-of-thought*). The explanations were generated via a state-of-the-art LLM able to handle multi-modal inputs (GPT-4). However, because the LLM can make errors, both the suggested diagnoses and explanations may sometimes be incorrect. **d** Our primary outcome is diagnostic accuracy, measured by the proportion of correct diagnoses per radiologist. To reflect clinical practice, diagnoses were collected as free-text responses (which is unlike the original *New England Journal of Medicine Image Challenge* using a multiple-choice format). As secondary outcomes, we assess how frequently radiologists adhere to correct LLM advice or overrode incorrect LLM advice.

Here, we analyzed the effect of different formats of LLM-generated explanations on the diagnostic accuracy of physicians (Fig. 1). To do so, we conducted a randomized experiment in which radiologists were tasked to diagnose patients based on clinical vignettes containing both text and radiological images. With 101 recruited radiologists and 2020 total assessments, our study substantially exceeds the sample sizes of related work in this domain (e.g.,^28–32^), but we emphasize the proof-of-concept character of this study and the need for further studies to demonstrate external validity. Using a between-subject design, we varied the *explanation format*. We used the following three treatments: (1) a *standard output* that offered a diagnosis without explanation; (2) a *differential diagnosis*, which outputted the five most likely differential diagnoses in descending order; and (3) a *chain-of-thought explanation*, which detailed the reasoning process behind the diagnosis. By comparing diagnostic accuracy across treatment groups, we can directly assess how different explanation formats affect clinical decision-making. The explanations were generated from the patient cases, involving the radiological images, using a state-of-the-art, multi-modal LLM (GPT-4; see Methods). We conducted a memorization test^33^, resulting in consistently low similarity scores between the model’s continuations and true case descriptions, indicating that GPT-4 had not memorized the patient cases (see details later). Additionally, a control group performed the task without LLM support. We examined how the explanation formats influenced the diagnostic accuracy of physicians. Furthermore, we assessed how often doctors followed LLM advice or overrode (in)correct diagnostic suggestions. The results show that chain-of-thought explanations led to the best overall diagnostic accuracy. We further analyzed the heterogeneity in the effect across physicians and patient cases. Based on the controlled conditions of our vignette study, we provide evidence-based insights for *how* to design explanations in medical LLMs that are of clinical utility.

## Results

### Experiment

To analyze how different formats of LLM-generated explanations affect diagnostic accuracy, we conducted a randomized experiment with 101 radiologists from the U.S. The radiologists had a mean of 13.6 years (standard deviation [SD] = 8.0) of medical experience. Further, the radiologists had different specializations, which we collected using a multi-option response format (e.g., general radiology: *n* = 57, abdominal imaging: *n* = 24, neuroradiology: *n* = 20, head and neck radiology: *n* = 9, cardiothoracic imaging: *n* = 8, and pediatric radiology: *n* = 5; see Supplementary Table 4). Detailed sociodemographic data for each study group can be found in Supplementary Table 28. Our sample comprised 101 radiologists, substantially exceeding the participant numbers in related studies of LLM-assisted clinical decision-making^28–32^.

Each radiologist was presented with 20 real-world patient cases (mean length: 55.4 words; SD = 18.8), each consisting of a brief clinical description and at least one radiological image (i.e., computed tomography [CT] or magnetic resonance imaging [MRI]), and was then asked to provide a diagnosis for the patient (*N* = 2020 assessments overall). Unlike the original *New England Journal of Medicine Image Challenge*, which uses a multiple-choice format, diagnoses were collected as free-text responses to better reflect clinical practice. For the same reason, patient cases were selected to cover a broad and diverse range of diagnostic challenges commonly encountered in radiology, following a principled inclusion process (see Methods). As a result, the cases span different radiological subspecializations (80% of patient cases can be answered using knowledge found in a standard textbook on diagnostic radiology^34^, while the remaining 20% require more specialized knowledge; see Supplementary Table 5). Of note, we deliberately selected both radiologists and patient cases from different subspecializations to later assess the diagnostic accuracy in general radiology as well as across subspecializations.

Participants were randomly assigned to either a control group with no LLM support or one of three treatment groups, each with a different form of LLM advice. By comparing diagnostic accuracy between the treatment groups, we can directly assess the effect of different types of explanations. In the three treatment groups, physicians received advice through different types of explanations generated by GPT-4, a state-of-the-art multi-modal LLM^35,36^. (1) *Standard output*. Here, a single best-guess diagnosis was provided, but the explanation is either absent, very brief, or paraphrasing the case description. (2) *Differential diagnosis*. Here, multiple plausible diagnoses were listed, each with a short justification, ordered by decreasing likelihood. This explanation format mirrors typical clinical reasoning, where multiple conditions are considered^37^. For the differential diagnosis format, we used two criteria for measuring diagnostic accuracy: top-1 diagnostic accuracy (the diagnosis is considered correct if the correct answer appears as the first item in the list) and top-5 diagnostic accuracy (the diagnosis is considered correct if it appears anywhere in the list of five). (3) *Chain-of-thought*. Inspired by best practice in LLM research^18^, an explanation was produced that provides a step-by-step rationale of how the LLM arrives at the final recommendation, which can give physicians a deeper understanding of the reasoning process and help them identify potential errors. All outputs were kept as generated, including any diagnostic errors or hallucinations, to mimic real-world LLM use in medical practice. Example outputs for each treatment group are shown in Fig. 1 The LLM-generated outputs exhibited substantial variations in length (i.e., the number of words in the output) across the different output formats (standard output: mean = 62.7, SD = 12.5; differential diagnosis: mean = 208.6, SD = 20.2; chain-of-thought: mean = 188.6, SD = 26.2; see Supplementary Figure 2).

To quantify the quality of LLM-generated diagnoses, we first evaluated the baseline diagnostic accuracy of the LLM output across all 20 patient cases. The LLM achieved moderate diagnostic accuracy overall (standard output: 75%; differential diagnosis: 65% for the top-1 answer and 80% for the top-5 answers; chain-of-thought: 80%; Supplementary Fig. 1), which underscores the need for careful evaluation of LLM outputs and which further implies that the selected patient cases were challenging for the model. To assess the benefits of different prompting strategies for generating correct LLM outputs, we estimated a logistic regression with conditions as independent variables. The LLM with chain-of-thought prompting performed better than the standard output (coef = − 0.288, 95% CI = [ − 1.779, 1.204], *P* = 0.705), and the LLM with chain-of-thought output performed similarly to the LLM with differential diagnosis output (coef = 0.000, 95% CI = [ − 1.549, 1.549], *P* = 1.000) (see Supplementary Table 6).

### Effect on diagnostic accuracy

**Fig. 2:**
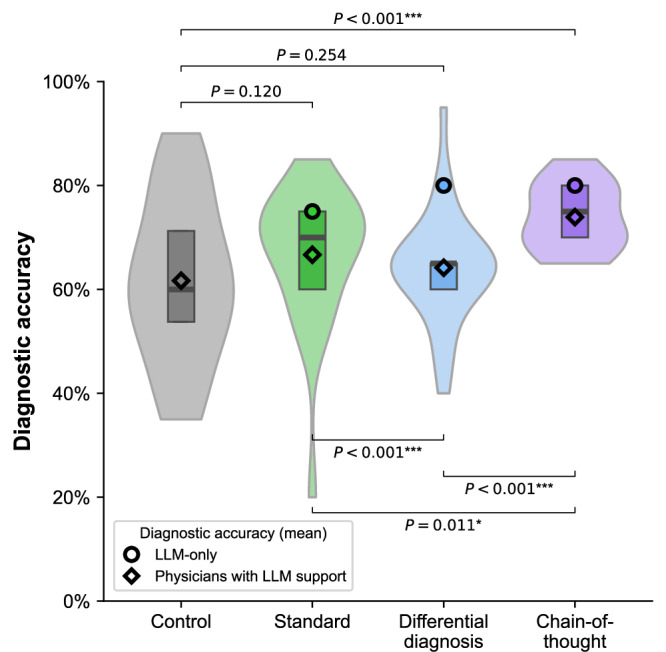
Diagnostic accuracy across different LLM explanations. The figure shows the distribution of diagnostic accuracy for all participants by condition as violin plots (which illustrate the probability density of the data). The boxplots within each violin indicate the 25% and 75% quartiles, with the median represented by the thick center line. For the differential diagnosis condition, the top-5 diagnostic accuracy is reported (the top-1 diagnostic accuracy is lower and corresponds to 65%). Statistical significance was assessed using one-sided Welch’s *t*-test to compare diagnostic accuracy between the treatment groups (in the main text, we report results from a regression analysis to obtain effect sizes).

Physicians augmented with LLM advice outperformed those in the control group without LLM advice (Fig. 2 for statistical comparisons based on one-sided Welch’s *t*-tests). To calculate effect sizes, we used an ordinary least squares (OLS) regression (Supplementary Table 7). Here, the diagnostic accuracy of physicians with standard output was comparable to the diagnostic accuracy of the control group (difference = 5.0 percentage points, 95% CI = [ − 1.8; 11.8], *P* = 0.150). Similarly, the diagnostic accuracy of physicians supported by a differential diagnosis was comparable to the control group (difference = 2.5 percentage points, 95% CI = [ − 4.0, 9.0], *P* = 0.446).

The format of the LLM advice has a large effect on diagnostic accuracy (Fig. 2 for statistical comparisons and Supplementary Table 7 for effect sizes). Physicians in the chain-of-thought group performed best with a 12.2 percentage point improvement over the control group (95% CI = [5.3, 19.2], *P* = 0.001). Separate comparisons for the chain-of-thought versus standard condition and chain-of-thought versus differential diagnosis condition showed improvements in the outcome measure by 7.2 percentage points (95% CI = [0.3, 14.2], *P* = 0.040) and 9.7 percentage points (95% CI = [3.2, 16.3], *P* = 0.004), respectively, which confirms that the chain-of-thought condition performed best (Supplementary Table 8).

Even after controlling for various physician-specific control variables such as years of medical experience, radiology-specific expertise, hours per week spent on visual inspections, IT skills, and experience with medical AI, the effects remain robust (see Supplementary Table 9 for the regression results). Physicians with chain-of-thought explanations (difference = 15.1 percentage points, 95% CI = [7.0; 23.2], *P* < 0.001) outperformed the control group (difference = 7.0 percentage points, 95% CI = [ − 0.9; 14.9], *P* = 0.081). Further, physicians supported by chain-of-thought explanations tend to perform better than the standard output group but only at the 10% significance level (difference = 7.0 percentage points, 95% CI = [ − 0.9; 14.9], *P* = 0.081), while the comparison with the differential diagnosis group was not statistically significant (difference = 3.9 percentage points, 95% CI = [ − 3.9; 11.7], *P* = 0.325).

The findings also hold when accounting for the decision time for all cases, the length of the LLM outputs, and the length of the answers (i.e., length measured by the number of words) entered by participants (see Supplementary 10). Physicians in the chain-of-thought group significantly outperformed the control group (difference = 4.6 percentage points, 95% CI = [1.0; 8.1], *P* = 0.012), whereas physicians in the differential diagnosis condition performed significantly worse than the control group (difference = − 4.8 percentage points, 95% CI = [ − 8.1; − 1.5], *P* = 0.005). While the standard condition did not differ significantly from the control group (difference = 2.1 percentage points, 95% CI = [ − 3.5; 7.6], *P* = 0.467), physicians in the chain-of-thought condition also outperformed those in the standard condition (difference = 4.6 − 2.1 = 2.5 percentage points). Further, a breakdown of the diagnostic accuracy by patient cases is in Supplementary Figure 3, confirming that case difficulty varies as intended based on our inclusion criteria.

To account for the baseline diagnostic accuracy across different prompting strategies, we estimated a logistic regression model that controlled for the correctness of the LLM diagnoses and the correctness of the LLM explanations. The standard output group and the differential diagnosis group were outperformed by the chain-of-thought group (coef = 0.504, 95% CI = [0.094, 0.914], *P* = 0.016; and coef = 1.014, 95% CI = [0.646, 1.383], *P* < 0.001; respectively) (see Supplementary Table 26). Additionally, we restricted our analysis to patient cases for which both the LLM’s final diagnosis and the explanation were uniformly correct or uniformly incorrect across all treatment groups. For example, we excluded cases where the model correctly diagnosed a patient under the chain-of-thought explanation but failed under the standard output, and vice versa. We find the standard output group (89.58%) had the lowest diagnostic accuracy (77.60%) compared to the differential diagnosis group (95.83%) and the chain-of-thought explanation (94.02%) (control group: 77.60%; Supplementary Fig. 4). While the procedure ensures that our analysis remains unbiased by differences in model performance arising from varying prompting strategies, it also excludes cases for which the support by LLMs made a larger difference on physicians’ decision-making, thus showing no significant effect from chain-of-thought explanations.

### Mechanism of adherence and overriding

**Fig. 3:**
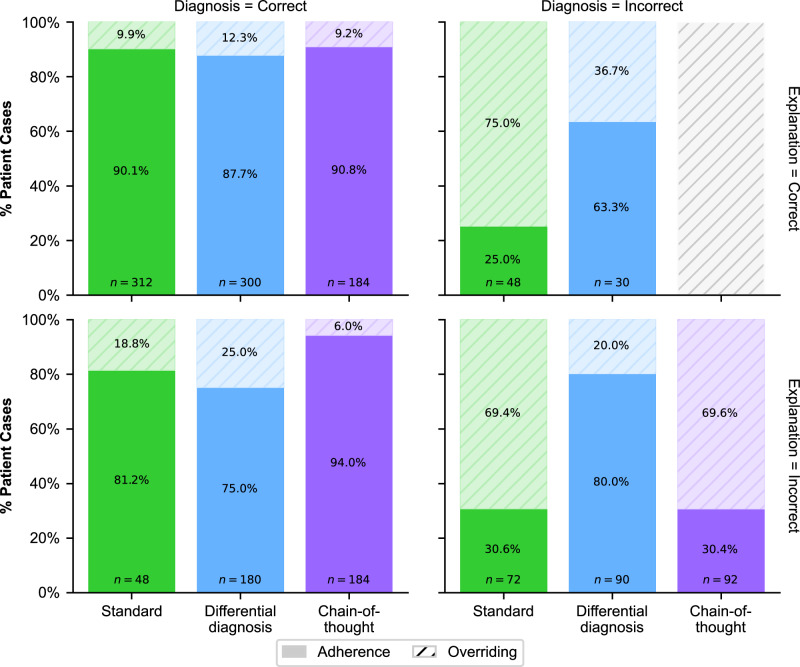
Adherence vs. overriding LLM advice. Breakdown of adherence for a correct/incorrect LLM diagnosis and for a correct/incorrect LLM explanation. Here, *adherence* was calculated as the proportion of patient cases in which participants' diagnoses matched the LLM-generated diagnoses. Correspondingly, *overriding* was defined as 1 − *adherence*. Reported is the adherence to the top-1 answer in the differential diagnosis explanation. Results for the top-5 answers are in Supplementary Fig. 11. Specifically, the figure illustrates physician adherence to LLM-generated output, conditioned on the correctness of the diagnosis (columns) and the explanation (rows). Each panel represents a specific condition of diagnosis and explanation correctness. Comparison of the left column (diagnosis = correct) with the right column (diagnosis = incorrect) reveals that diagnostic correctness influences adherence, particularly revealing over-adherence to differential diagnoses when the recommended diagnosis is incorrect. In contrast, comparing the top row (explanation = correct) with the bottom row (explanation = incorrect) suggests that the correctness of the explanation has a limited impact on adherence across different advice types. These observations indicate that hallucinations in the diagnosis are a stronger driver of potentially inappropriate adherence than hallucinations in the explanation. Note that the condition of correct explanation and incorrect diagnosis is not represented due to a lack of generated data for this scenario, which should be expected given that chain-of-thought prompting is designed to generate an explanation that matches a given diagnosis.

We explored adherence and overriding behavior, two terms commonly used in literature on human-computer interaction to describe how participants followed correct or incorrect LLM output (Fig. 3). For differential diagnosis, we assessed adherence based on the top-1 recommendation (results for top-5 recommendations are consistent; see Supplementary Fig. 11). When the LLM-generated diagnosis was incorrect (Fig. 3, right column), adherence was highest in the differential diagnosis group irrespective of the correctness of the explanation (adherence for incorrect diagnosis but correct explanation: 63.3%; adherence for incorrect diagnosis and incorrect explanation: 80.0%), which also exceeded that of the standard output group (25.0% and 30.6%, respectively) and the chain-of-thought group (adherence for incorrect diagnosis and incorrect explanation: 30.4%). Note that, in the chain-of-thought group, there are no cases with an incorrect diagnosis accompanied by a correct explanation, which is expected, given the structure of the prompting strategy^18^, which aimed to generate the explanation as a rationale leading to the final diagnosis. Thus, physicians in the differential diagnosis group appeared more inclined to follow the LLM-generated advice even when it was wrong.

In contrast, when the LLM-generated diagnosis was correct (Fig. 3, left column), adherence was highest in the chain-of-thought explanations (adherence for correct explanation: 90.8%; for incorrect explanation: 94.0%), leading to more appropriate reliance on the LLM-generated advice. Here, adherence was higher compared to both the standard output group (90.1% and 81.2%) and the differential diagnosis group (87.7% and 75.0%). In sum, physicians supported with differential diagnosis explanations had consistently high adherence, even when the LLM was incorrect and thus often failed to override when the LLM advice was wrong. Conversely, the chain-of-thought explanation (in comparison to the differential diagnosis) led to more selective adherence, with physicians being more likely to follow correct LLM advice and to override incorrect LLM advice.

### Heterogeneity across physicians and patient cases

**Fig. 4:**
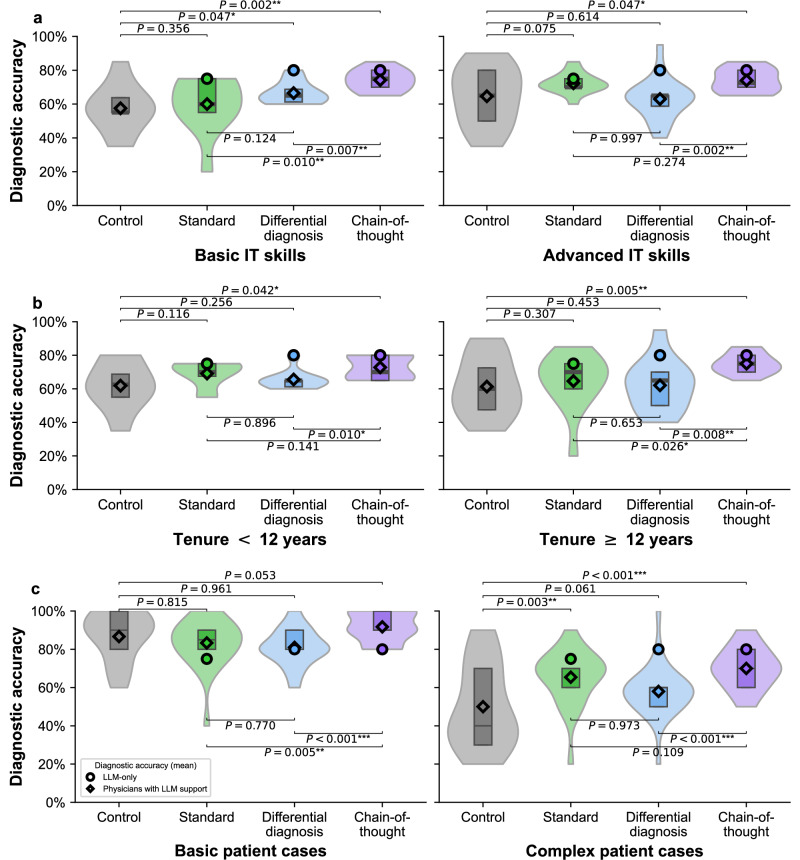
Heterogeneity across physicians and patient cases. **a** Diagnostic accuracy was compared between different levels of IT skills, as defined by a post-task survey. Participants were classified as having advanced IT skills (*n* = 56) when reporting “very good'', while all others were classified as having basic IT skills (*n* = 43). **b** Diagnostic accuracy was compared across different numbers of years with medical expertise in radiology (*n* = 46 have a tenure of < 12 years, while *n* = 53 have tenure of ≥ 12 years). **c** Diagnostic accuracy for basic and complex patient cases was assessed by dividing cases into two equally sized subsets based on the median diagnostic accuracy observed in the control group, resulting in two subsets with each 10 patient cases. As such, the cases are split into subgroups based on the median diagnostic accuracy of the control group and are thus formed independently of the difficulty rating from the radiologists in our panel. The boxplots within each violin indicate the 25% and 75% quartiles, with the median represented by the thick center line. Statistical significance was assessed using one-sided Welch’s *t*-test. Estimated effect sizes using regression analysis are in Supplementary Tables 12 to 16.

To examine heterogeneity across physicians, we analyzed diagnostic accuracy across physician subgroups with different IT skills and medical tenure (Fig. 4a, b). Overall, the chain-of-thought group tends to achieve a higher diagnostic accuracy than both the control and the differential diagnosis groups. A similar trend was observed when accounting for the complexity of patient cases (Fig. 4c).

### Heterogeneity across general vs. specialized radiologists

**Fig. 5:**
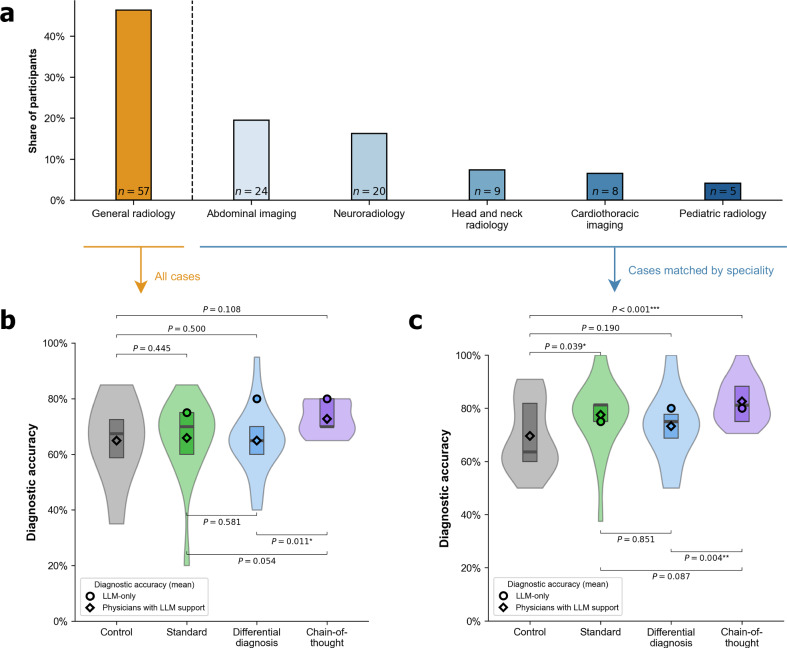
Diagnostic accuracy across different radiological backgrounds (general radiologists vs. radiologists with subspecializations). **a** Our participant sample includes general radiologists and radiologists from different subspecializations, collected via a multi-option response format (see Supplementary Table 4). Because participants could select multiple specializations, individuals may appear in more than one subspecialty. We then matched patient cases to the radiologists based on their background, as follows: **b** The plot shows the diagnostic accuracy assessed across all 20 patient cases for the sample of *n* = 57 general radiologists. **c** The plot shows diagnostic accuracy for the subgroup of specialized radiologists (*n* = 66), evaluated only on patient cases matching their subspecialty. The assignment of patient cases to subspecialty is in Supplementary Table 5. The boxplots within each violin indicate the 25% and 75% quartiles, with the median represented by the thick center line. Statistical significance was assessed using one-sided Welch’s *t*-test. Estimated effect sizes using regression analysis are in Supplementary Tables 18 and 19.

To explore the effect of explanation formats across different radiologist backgrounds, we grouped participants into general radiologists and those with subspecialization (see Fig. 5a and Supplementary Table 5). Then, we compared diagnostic performance in general radiologists (*n* = 57) against the diagnostic performance in participants with subspecialization (*n* = 66; note that participants can choose more than one area of specialization), but where we selected cases that matched the corresponding expertise.

While subspecialization is more common in certain regions such as the United States, many radiologists around the world continue to practice as generalists, and general radiology remains widespread in a variety of clinical settings^38–40^. We thus evaluate the diagnostic accuracy for general radiologists on all cases in our sample (Fig. 5b). Here, the diagnostic accuracy in the chain-of-thought group tends to be higher than in the standard output group but the improvement is only marginally significant (*P* = 0.0504). Interestingly, the diagnostic accuracy in the chain-of-thought group is again significantly higher than in the differential diagnosis group (*P* = 0.011) (see Supplementary 19 for regression results).

We next evaluate the diagnostic accuracy for specialized radiologists based on the patient cases matched by their subspecialization (Fig. 5c). While the difference between the differential diagnosis group and the standard output group was not significant (*P* = 0.129), we find that specialists in the chain-of-thought condition performed significantly better than in the control condition (*P* = 0.001) and in the differential diagnosis condition (*P* = 0.003). To understand the benefit of different explanation formats in specialized diagnostic tasks, we again performed a regression analysis (Supplementary 18). Specifically, we estimated the effect size of different explanation formats on the diagnostic accuracy for patient cases that matched the specific subspecialty of radiologists. Relative to the control group, improvements in diagnostic accuracy were observed for the standard output group (coef = 0.079, *P* = 0.0566) and the chain-of-thought group (coef = 0.130, *P* = 0.003) conditions. In contrast, the differential diagnosis condition showed no significant effect (coef = 0.037, *P* = 0.351). In other words, the benefit of chain-of-thought explanations over differential diagnosis explanations is not driven by radiologists’ subspecialty expertise, but is robust even for specialized radiologists.

## Discussion

The rapid adoption of LLMs in clinical settings^41^ raises the question of how physicians and LLMs can most effectively collaborate to improve patient outcomes. While LLMs can help physicians verify their advice by providing explanations, the best-suited design of such explanations remains unclear. Our experiment demonstrates that the *format* of explanation plays a surprisingly important role in the effectiveness of LLM support. In contrast to prior research demonstrating that LLM-augmented physicians ("human-in-the-loop”) generally outperform those without LLM support^4,7,12,15,42^, we find substantial variation in the effectiveness of LLM support depending on how the information is presented. Interestingly, under the controlled conditions of our vignette study, chain-of-thought explanations seem to be associated with larger performance gains than presenting differential diagnoses (*P* < 0.001 in Fig. 2; *P* = 0.001 and *P* = 0.003 in Fig. 5b,c, respectively). Overall, the effects were largely consistent across physicians with varying levels of medical experience and IT proficiency, as well as for general radiologists and those with subspecialization.

The key mechanism behind the performance gain from chain-of-thought explanations appears to be the physicians’ improved ability to assess the correctness of LLM output based on the provided reasoning. When LLMs provide step-by-step reasoning, physicians can better assess whether to follow the model’s advice (appropriate AI reliance) or override flawed suggestions (appropriate self-reliance)^43^. This mechanism aligns with research in cognitive psychology: structured reasoning enhances judgment and decision-making by making underlying thought processes transparent^44^. Our findings specifically suggest that the observed gains in physician accuracy are associated with how the explanation format supports critical evaluation of the model’s output. Differential diagnosis explanations, in contrast, may induce excessive adherence to LLM advice even when incorrect. This pattern reflects automation bias. McDuff et al. found that physicians exposed to LLM-generated differential diagnoses sometimes performed worse than the LLM alone^42^, but they did not test whether chain-of-thought explanations might mitigate this problem. Our findings suggest they do: when LLMs have imperfect accuracy, chain-of-thought explanations support critical evaluation more effectively than differential diagnosis formats.

The two explanation formats differ not only in reasoning depth but also in how many diagnoses they present. Chain-of-thought reasoning focuses on a single diagnostic pathway, leaving physicians free to consider alternatives when they detect inconsistencies. Differential diagnoses, by contrast, enumerate multiple options—here, five possibilities—without providing explicit reasoning for each. This enumeration may constrain the physician’s hypothesis space: when presented with named options, clinicians may restrict their consideration to the listed diagnoses, reducing their sensitivity to alternatives even when none of the presented options fully satisfies the clinical picture. Research focused on Fischhoff’s fault tree (which studies how people judge the completeness of branching decision diagrams) suggests this effect strengthens with more enumerated options: presenting five diagnoses creates a stronger illusion of completeness than presenting fewer, making physicians less likely to consider omitted alternatives^45^.

Taken together, chain-of-thought explanations improve physician performance by providing transparent reasoning that enables critical evaluation of LLM advice, whereas differential diagnoses may constrain physicians’ diagnostic reasoning by presenting a fixed list of options without explicit justification. Notwithstanding, the differential diagnosis format may be appropriate when the LLM accuracy is high and, thus, could be more relevant in future LLMs with overall higher baseline diagnostic accuracy. Here, it would be interesting to explore how careful physician training may help reduce existing nonadherence and overriding behavior. Finally, because the perceived helpfulness was similar across treatment groups (Supplementary Fig. 6), the benefits from LLM advice seem unrelated to subjective factors or preferences. Instead, the benefits appear to originate from how the explanation format enables physicians to more effectively evaluate LLM advice and incorporate it into their own decision-making processes.

In general, prompt design is known to influence the correctness of LLM outputs^18,46^. In our study, diagnostic accuracy varied with the prompting strategy: the chain-of-thought strategy improved the accuracy of the LLM output by 5 percentage points relative to a naïve strategy with a default prompt and thus no prompt tuning. This finding aligns with best practice recommendations^18,47^ and underscores the importance of carefully fine-tuning prompts for clinical applications. Another implication of this finding is the need to boost AI literacy among physicians to help them design effective prompts, especially in light of the fact that the standard strategy for LLM deployment is one without formal training in prompt strategies^48^. The timing of AI advice presentation may also matter. Research on diagnostic decision support shows that providing suggestions before physicians form initial hypotheses significantly improves accuracy, while late suggestions (after hypothesis formation) show minimal benefit^49^. This suggests LLM explanations may be most effective when presented early in the diagnostic process. Further, while the varying levels of diagnostic accuracy across different prompting strategies may have initially complicated direct comparisons, this was done intentionally and is a key strength of our study because we mimic a real-world scenario in medical LLM deployment. Nonetheless, our robustness checks confirmed that the effect of explanation formats remained robust even after adjusting for the diagnostic accuracy of the prompting strategies (see Supplementary Table 26). Notably, our main results and robustness checks consistently show that the language model alone is outperforming all groups of radiologists.

Previous medical research has primarily focused on comparing the diagnostic accuracy between physicians augmented with LLM support versus those without it^4,7,12,15^, yet without isolating the effect of prompt design. As a result, the impact of explanation formats on diagnostic decision-making has remained unclear. Even though the importance of *how* information is presented in medical AI applications is increasingly acknowledged^50–52^, empirical research focusing on decision-making is limited. Some work has explored visual annotations generated by AI tools to assist in medical image interpretation^53–56^, but these studies primarily address search-and-locate tasks (e.g., lesion detection) and use outputs different from ours (e.g., heatmaps). In contrast, our study examines how different LLM explanation formats can improve diagnostic decisions.

This study has multiple limitations that present interesting directions for future research. First, we focused on only one medical specialty; however, diagnostic decisions are central to radiology and common in routine medical practice^57,58^. Moreover, the set of patient cases covers a broad range of typical radiological assessments. With 101 recruited radiologists and 2020 total assessments, our study substantially exceeds the sample sizes of related work in this domain (e.g.,^28–32^). Future research could explore the extent to which our findings extend to other medical specialties. Second, our analysis focused on a single time point; longitudinal studies are needed to analyze the long-term effect of LLM assistance on medical practice. Third, while we evaluated diagnostic accuracy, we did not assess the potential harm that can arise from incorrect decisions or other patient outcomes. Fourth, although some physicians in the control group reported having accessed the Internet analogous to routine practice, none indicated using LLMs, suggesting that the risk of conflated results is low. Fourth, the effects of the increased performance from chain-of-thought explanations remained statistically significant after controlling for decision time. Future LLMs may generate even more informative explanations, but we also acknowledge the inherent limitations of LLMs, such as their tendency to produce hallucinations or permeating biases^59,60^. The risk of hallucinations warrants future research on uncertainty quantification methods for language models. The need for reliable uncertainty quantification is further amplified by the observation that explanations are found to be unfaithful to a model’s decision and thus are of little help to verify a model’s reasoning process^61^. Notwithstanding, the real-world implementation of LLM in medical settings requires a careful approach considering potential risks, and we thus leave evidence from field experiments, necessary to establish ecological validity in routine care, to future research, as our primary goal here is to stimulate discussion about explanation formats.

The study employed a randomized between-subjects design in which different groups of physicians were exposed to different explanation formats. While randomization and extensive robustness analyses controlling for physician experience, IT familiarity, and case characteristics mitigate concerns about systematic group differences, unobserved variation in physician skill or chance cannot be fully ruled out. As a result, although the findings strongly suggest that the chain-of-thought explanation format contributed to improved diagnostic performance, future studies using within-subject or crossover designs—while carefully addressing potential learning and carryover effects—could further strengthen causal effect estimation.

Our results are based on GPT-4, a state-of-the-art multi-modal LLM for which the diagnostic accuracy closely matches that of clinical experts^6,62,63^. We further selected patient cases from the *New England Journal of Medicine Image Challenge* because these are expert-curated, widely recognized for their educational value, and frequently used in prior research evaluating medical LLMs^64–67^. Still, a potential limitation is the possibility that some cases may have been part of the training data for GPT-4. However, the diagnostic accuracy of GPT-4 on our task is moderate and includes frequent errors, suggesting that GPT-4 did not simply memorize the patient cases, especially as the original dataset does not include detailed explanations. Prior work also shows that small data contamination has only minimal impact on benchmark validity^68–70^, and evaluating LLMs on publicly available datasets is standard practice^64,71^. Nevertheless, while we report comparisons *between* LLM-supported physicians and the control group for completeness, our main findings rely on comparisons *within* LLM-supported conditions where explanation format—not data overlap—is responsible for observed effects.

Up to this date, misdiagnosis is a common issue in medical practice^9^, including in radiology^57,58^. While LLMs hold promise for reducing diagnostic errors^8^, designing systems that effectively support collaboration with physicians remains challenging. Our findings show that, under the controlled conditions of our vignette study, the format of LLM explanations has a large impact on decision performance, thereby highlighting the need for a human-centered approach to designing real-world LLM implementations in order to offer clinical utility and ultimately improve patient outcomes. Future LLMs may provide even more informative explanations and thereby further improve diagnostic accuracy.

## Methods

This work analyzes how the diagnostic accuracy of physicians is affected by different explanation formats of LLMs. We preregistered our hypotheses (see https://aspredicted.org/4tgb-sr3z.pdf) and tested them in a randomized experiment using a between-subject design. We complied with all local ethical regulations. The research design was approved by the Ethics Commission of LMU Munich (EK-MIS-2024-320). All participants provided informed consent.

### Procedure

To evaluate the effect of different explanation formats on the diagnostic accuracy of physicians, we designed the following experiment. First, participants received information about the study objectives. After giving informed consent, each participant was introduced to the diagnostic task and randomly assigned to one of four groups: a control group (*n* = 24) or one of three treatment groups—namely the standard output group (*n* = 24), the differential diagnosis group (*n* = 30), or the chain-of-thought group (*n* = 23). Only participants in the treatment groups received LLM-based advice. Once the participants completed the diagnostic tasks, they were asked to fill out a post-task survey. The survey collected socio-demographic information (e.g., experience in medicine in years, experience specifically in radiology in years) and asked general questions about the task (e.g., helpfulness of the LLM support). A full list of the survey items is in Supplementary Table 2.

Each participant was shown 20 cases and then asked to provide a diagnosis in the form of an open-ended text. This approach differs from the original *New England Journal of Medicine Image Challenge*, which uses a multiple-choice format. However, we opted for free-text responses to better reflect clinical practice, where diagnoses have to be generated without predefined answer options. The responses were manually coded by the author team to correct for minor typos. The cases were shown in random order to minimize bias from potential learning effects or fatigue. Each case consisted of a descriptive text plus at least one (and sometimes two) radiological images (i.e., computed tomography [CT] or magnetic resonance imaging [MRI]). Participants completed the tasks without a maximum time limit (a minimum time limit of 10 seconds was enforced for each task to ensure high-quality answers). After completing the diagnostic tasks, participants filled out a post-task survey that included an embedded attention check (a directed-response item asking participants to select a specific answer).

Our experiment was designed to reflect the diversity and heterogeneity of real-world radiology practice. To that end, we included both general radiologists and those with various subspecializations (Supplementary Table 4) and selected patient cases relevant to general radiology as well as cases spanning multiple radiological subspecialties (Supplementary Table 5). This design allowed us to assess diagnostic accuracy across different radiological backgrounds, i.e., both in general radiology and in cases where physicians engaged with content aligned with their specific subspecialty expertise.

### Patient cases

All patient cases included in this study were sourced from the *New England Journal of Medicine Image Challenge*^72^, which is a high-quality collection of peer-reviewed cases, including both common and rare diagnoses, and which is used in related research to assess the diagnosis assistance from LLMs^42^. Each case consists of a short text describing the patient’s characteristics and symptoms, along with at least one medical image, such as photographs of dermatological findings, endoscopic images, histopathological slides, or radiological images. Overall, the patient cases cover both general radiology (80% of patient cases can be answered using knowledge found in core radiology educational handbooks^34^) as well as different subspecializations (20% of the patient cases require specialized expertise; see Supplementary Table 5).

In the first step, a set of only radiological cases and images were randomly sampled and the difficulty of diagnosing each case was rated by a panel of three radiologists (J.Ru., S.S., and B.F.H.) on a 5-point Likert scale. Of those, we selected 20 cases to reflect a broad spectrum of possible diagnoses and levels of difficulty, but omitted cases that were rated as either too easy or too difficult (e.g., very rare diagnoses only based on case reports). This ensured a broad and diverse set of diagnostic challenges commonly encountered in radiology. Of the 20 selected cases evaluated using a five-point Likert scale (1 = easy, 5 = difficult), 8 cases were rated as difficulty level 1, 5 cases as level 3, 5 cases as level 4, and 2 cases as level 5. Simpler patient cases included, e.g., disseminated brain metastasis or hepatic echinococcosis, while more complex cases included, e.g., disseminated mycobacterium avium complex (MAC) infection or infantile hepatic hemangiomas. The difficulty of selected cases is also reflected by the low baseline diagnostic accuracy of our control group. As such, the diversity of our patient cases is intended to mirror medical practice.

In the second step, we conducted a memorization test^33^ to verify that GPT-4 had not been exposed to our patient cases during training. Thus, we truncated each case at five evenly spaced intervals and prompted the model to continue the patient description. We then compared the generated continuations against the original text using two complementary similarity metrics: the Ratcliff/Obershelp sequence matching ratio^73^ and the BLEU score^74^. Average similarity scores across all patient cases were consistently low (Ratcliff/Obershelp: 0.049, BLEU: 0.006). As a baseline comparison, we tested memorization on medical passages from Wikipedia (i.e., content likely seen during training), which yielded substantially higher BLEU scores (0.048), demonstrating an 8-fold increase compared to our evaluation cases. The low similarity scores suggest that the patient cases were not memorized.

The median word count of the texts was 54.5 words, with small variability (minimum: 24 words; maximum: 85 words). Of the 20 cases, 15 include one radiological image, while the remaining 5 include two.

### LLM advice

Participants in the treatment groups received advice generated by an LLM (GPT-4, version 2024-02-15-preview, temperature = 0.7, accessed via Microsoft Azure) based on the patient’s text and imaging data. We followed best practice in prompt design^18,46,47^ to produce different output formats depending on the treatment groups (see Supplementary Table 1 for the exact prompts):

1. *Standard output* presents a single diagnosis along with either no explanation or only a concise rationale that typically mirrors the input prompt. This approach reflects LLM use cases in which no custom prompting strategy is employed. Furthermore, it aligns with the format commonly adopted in benchmarking studies from LLM research^5,12^, where the focus is primarily to assess the diagnostic accuracy of the LLM.
2. *Differential diagnosis* lists multiple possible diagnoses (here: the top five diagnoses) ordered by likelihood from most likely to less likely. This output format is motivated by that differential diagnosis is a common approach in routine medical practice^16,17^ and further acknowledges that LLMs should perform better when the top-*k* predictions are considered rather than the top-1 prediction. Intuitively, such a differential diagnosis should help physicians consider alternative diagnoses, especially for rare diseases. Unless stated otherwise, we report diagnostic accuracy for the top-5 answers, meaning that the differential diagnosis was considered correct if the correct diagnosis was contained in one of the five LLM-suggested diagnoses. Following this logic, top-1 considers the differential diagnosis correct if the *first* answer is correct. Note that the LLM does not calculate explicit probabilities for ordering the potential diagnosis; the ranking likely reflects patterns in the model’s training data (see Discussion for further details on this limitation). Our design choice to list *k* = 5 diagnoses in the differential diagnosis format was guided by clinical reasoning to offer a reasonable tradeoff between cognitive load for physicians and potential gains in diagnostic accuracy (see also ref. ^42^ for a quantitative analysis supporting this choice). This decision is further supported by the distribution of correct answers: the correct diagnosis appeared as the top-1 suggestion in 13 out of 20 cases (65%) and within the top-2 suggestions in 16 out of 20 cases (80%), while the diagnostic accuracy remained stagnant thereafter. This pattern suggests that increasing the number of suggestions further would offer minimal incremental benefit while potentially introducing unnecessary complexity for physicians.
3. *Chain-of-thought explanation* provides a detailed, step-by-step description of the reasoning process behind how the diagnosis is made. Intuitively, this is motivated by that, on the one hand, research in machine learning suggesting that chain-of-thought prompting can improve performance^18^ and, on the other hand, such format should help physicians in comparing the diagnostic process against their domain knowledge and thus identify—and eventually—override errors in the LLM advise.

We selected GPT-4 (version: 2024-02-15-preview) due to its state-of-the-art performance for diagnostic reasoning capabilities^6,12,13^. Further, GPT-4 is designed to handle multi-modal inputs, which allows us to process both textual descriptions and radiological images. A comparison regarding the diagnostic accuracy for another LLM is in Supplementary Fig. 12, which suggests that, while other LLMs perform similarly overall, these are slightly inferior.

### Outcome

To evaluate the effect of different explanation formats, the primary outcome for our study was the *diagnostic accuracy* at the physician level. Specifically, we mapped the answers onto a dichotomous value ( = 1 if the diagnosis is correct and 0 otherwise) and then averaged the values across all 20 patient cases for each participant. When determining whether answers were correct, we only included fully correct answers. In an additional analysis, we looked at answers, which were partially correct, but missing important details for full diagnosis (e.g., in case 2, “metabolic toxicity” is partially correct, but the underlying cause of “manganese poisoning” is missing; or, in case 3, where “gastric outlet” is partially correct, but the “superior mesenteric artery (sma) syndrome” as the cause of the obstruction is missing) and performed a robustness check where such partially correct responses were counted as correct and arrived at similar findings (see Supplementary Fig. 5).

We also collected additional measures through a post-task survey to assess the perceived interactions with the LLM support (e.g., helpfulness). The survey items are listed in Supplementary Table 2, which we used to analyze the perceptions of different explanations (see Supplementary Figs. 6 to 10).

### Study participants

The study was conducted online (November 21, 2025 – December 1, 2025) using the online platform Qualtrics (Qualtrics International Inc.). We recruited physicians specializing in radiology from the U.S. via MSI-ACI (a market research firm with designated panels of medical professionals)^75^. Participants were compensated by MSI-ACI. No additional performance-based financial incentives were provided to preserve the ecological validity of the study and minimize potential biases due to extrinsic motivations, thereby mimicking the incentive structure of decision-making in medical practice^54,56^.

Participants were required to meet the following inclusion criteria: They had to be licensed physicians specialized in radiology, consent to the study, and have access to a device with internet access to participate in the study. Following pre-registered exclusion criteria, we then excluded participants who did not complete the study and failed the attention check.

A total of 156 participants consented to join the study, of whom 101 completed the tasks and met the inclusion criteria, which thus forms the final sample for analysis. The reasons for exclusion were incomplete participation (*n* = 42), failure to pass the attention checks (*n* = 11), or duplicate entries (*n* = 2). The radiologists have different specializations, which we collected using a multi-option response format (e.g., general radiology: *n* = 57, abdominal imaging: *n* = 24, neuroradiology: *n* = 20, head and neck radiology: *n* = 9, cardiothoracic imaging: *n* = 8, and pediatric radiology: *n* = 5; see Supplementary Table 4).

### Statistical analysis

To compare the diagnostic accuracy across different conditions, we used analyses of variance (ANOVA) and one-sided Welch’s *t*-tests. This approach was selected due to its robustness against unequal variances and different sample sizes between groups. A significance level of *α* = 0.05 was used for all statistical comparisons.

To calculate effect sizes comparing the explanations, we estimated an OLS regression model at the physician level. The outcome was the diagnostic accuracy, modeled as a function of explanation type, with the control group as the reference and separate variables to capture the effects for groups supported by standard output, differential diagnosis, and chain-of-thought explanations. To estimate the effect of different prompting strategies on both the diagnostic accuracy of the LLM-only approach (without human involvement), we estimate a logistic regression at the assessment level. We report the coefficients on the log-odds scale for reasons of comparability.

All analyses were conducted in Python (3.11.11) using the packages numpy (2.2.1), pandas (2.2.3), scipy (1.15.1), pymer4 (0.8.2), and statsmodels (0.14.4). The data visualizations were created with seaborn (0.13.2) and matplotlib (3.10.0).

### Robustness checks

We conducted an extensive series of robustness checks to ensure the validity and reliability of our findings. (1) We repeated the regression models while incorporating various participant-specific controls to benefit from increased power (Supplementary Table 9). (2) We further controlled for the length of participants’ answers (in the number of characters), the length of the LLM-generated advice (in the number of words), and the average duration of participants to complete the 20 patient cases (in minutes). The analysis results are shown in Supplementary Table 10. (3) To mitigate the influence of extreme outliers in our regression, we winsorized the diagnostic accuracy of study participants by limiting the extreme values in the data to the 5th and 95th percentiles (Supplementary Table 20). (4) To account for diagnostic ambiguity (e.g., when a diagnosis appears accurate but omits a specific sub-diagnosis necessary for determining the appropriate treatment strategy), our initial coding scheme classified partially correct responses as incorrect. We now repeated the analysis and counted such responses as correct (Supplementary Fig. 5). (5) We estimated a mixed-effects model to control for heterogeneity across physicians and tasks (see Supplementary Table 21). (6) We estimated a quasi-binomial regression, which may limit the interpretability of the estimated effect sizes but which can better accommodate for the fact that the diagnostic accuracy is in the range between 0% and 100% (see Supplementary Table 22). (7) We controlled for the characteristics of radiological images provided in the patient cases, including the number of images in the case, the source of the image (i.e., CT or MRI), and the body region displayed in the image (i.e., abdomen, head, spine, or thorax) (see Supplementary Table 27). Across all robustness checks, the findings remained consistent with the main results.

In principle, different prompting strategies can influence the accuracy of LLM outputs. While such variability reflects real-world LLM use, potential differences in accuracy across different prompting strategies may decline with future LLMs. Thus, to remove bias from different prompting strategies, we further controlled for the diagnostic accuracy of the LLM advice within each treatment arm, which allows us to yield “ceteris paribus” estimates by factoring out variability in LLM performance (Supplementary Table 26). The approach yielded qualitatively similar conclusions.

To compare the diagnostic accuracy between general radiologists and those with subspecialization, we conducted three additional analyses: (1) We repeated the above regression analysis while controlling for the subspecialization of radiologists (see Supplementary 17). (2) We focused our analysis on participants who self-reported to be general radiologists and evaluated the diagnostic accuracy for this subset of participants but using all patient cases (see Supplementary 19). (3) We evaluated the diagnostic accuracy of specialized radiologists based on patient cases that match their expertise (see Supplementary 18). In all analyses, the chain-of-thought explanation resulted in the largest improvements in diagnostic accuracy.

## Supplementary information

Supplementary Information

## Data Availability

De-identified data to reproduce the results is publicly available at https://github.com/PhilippSpitzer/LLM-Explanations-for-Radiology.

## Code availability

Code to reproduce the results is available via our GitHub at https://github.com/PhilippSpitzer/LLM-Explanations-for-Radiology. The GitHub also includes the survey of the online experiment as a Qualtrics .qsf file.
