# Supplementary material for: The effect of medical explanations from large language models on diagnostic accuracy in radiology
Source: NPJ Digit Med. 2026 Apr 23;9:333. doi: 10.1038/s41746-026-02619-0 (PMC13106719; doi:10.1038/s41746-026-02619-0)
Supplement: Supplementary file 1 — Supplementary Information [file 41746_2026_2619_MOESM1_ESM.pdf]

Supplementary information

Philipp Spitzer[1, ‡], Daniel Hendriks[1, ‡], Jan Rudolph[2], Sarah Schlaeger[2], Jens Ricke[2], Niklas Kühl[3,4†], Boj Friedrich Hoppe[2,5†], and Stefan Feuerriegel[*,5,6,†]

[1]Karlsruhe Institute of Technology, Karlsruhe, Germany

[2]Department of Radiology, LMU University Hospital, LMU Munich, Munich, Germany

[3]University of Bayreuth, Bayreuth, Germany

[4]Fraunhofer FIT, Bayreuth, Germany

[5]Munich Center for Machine Learning, Munich, Germany

[6]LMU Munich, Munich, Germany

[‡]Joint first author

[†]Joint last author

[*]Corresponding author: feuerriegel@lmu.de

Supplementary Figures

1 Baseline diagnostic accuracy of LLM advice 4
2 Length of LLM-generated explanations 5
3 Diagnostic accuracy across patient cases 6
4 Subset analysis for patient cases with LLM advice of equal diagnostic accuracy across conditions 7
5 Robustness check for the diagnostic accuracy when counting partial responses as correct. 8
6 Effect on helpfulness 9
7 Effect on usefulness 10
8 Effect on ease of use 11
9 Effect on trust 12
10 Effect on task load 12
11 Adherence vs. overriding LLM advice for different explanation formats 13
12 Comparison of GPT-4 vs. Claude 14

Supplementary Tables

1 Prompts for generating explanations 15
2 Items in the post-task survey 16
3 Patient cases included in the study 19
4 Radiologists by subspecialization. 21
5 Mapping of patient cases to subspecialization. 22
6 Diagnostic accuracy of the LLM across different prompting strategies 23
7 Effect of explanation types on diagnostic accuracy over the control group 24
8 Benefit of chain-of-thought explanations on diagnostic accuracy 25

9 Extended OLS regression of diagnostic accuracy with physician-level controls . . . 26
10 Extended OLS regression of diagnostic accuracy with advice-level controls. 27
11 Subgroup analysis for participants with basic IT skills 28
12 Subgroup analysis for participants with advanced IT skills 29
13 Subgroup analysis for participants with short tenure 30
14 Subgroup analysis for participants with long tenure 31
15 Subgroup analysis for basic patient cases 32
16 Subgroup analysis for complex patient cases 33
17 OLS regression controlling for radiologist subspecialization. 34
18 OLS regression explaining diagnostic for patient cases within participants' subspecialties. 35
19 OLS regression for diagnostic accuracy among general radiologists. 36
20 Robustness checks controlling for outliers 37
21 Mixed-effects model 38
22 Quasi-binomial regression 39
23 Effect on decision time (compared to control group) 40
24 Effect of decision time (compared to chain-of-thought) 41
25 Logistic regression at the assessment level 42
26 Logistic regression controlling for correct diagnosis and correct explanation in the LLM advice 43
27 Regression controlling for the characteristics of radiological images 44
28 Demographics of study sample by condition. 45

Supplementary Figures

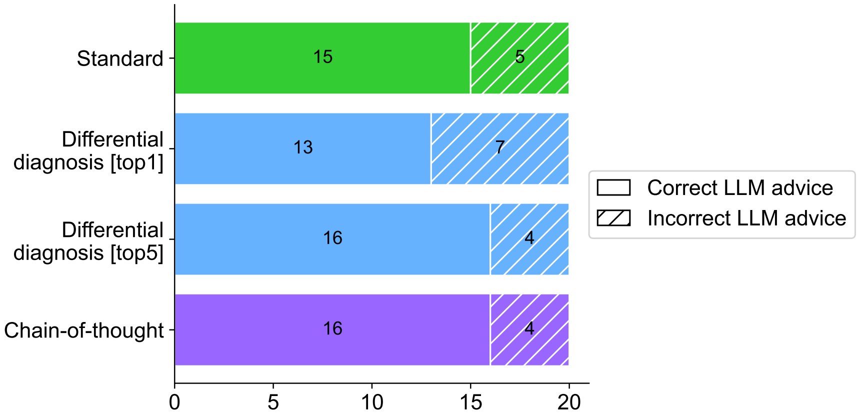

Supplementary Figure 1: **Baseline diagnostic accuracy of LLM advice.** The diagnostic accuracy reports how often the LLM advice was stating the correct diagnosis (regardless of whether the explanation was correct or not). For the differential diagnosis, top-1 refers to whether the first answer was correct, while top-k refers to whether any of the five answers contained the correct diagnosis.

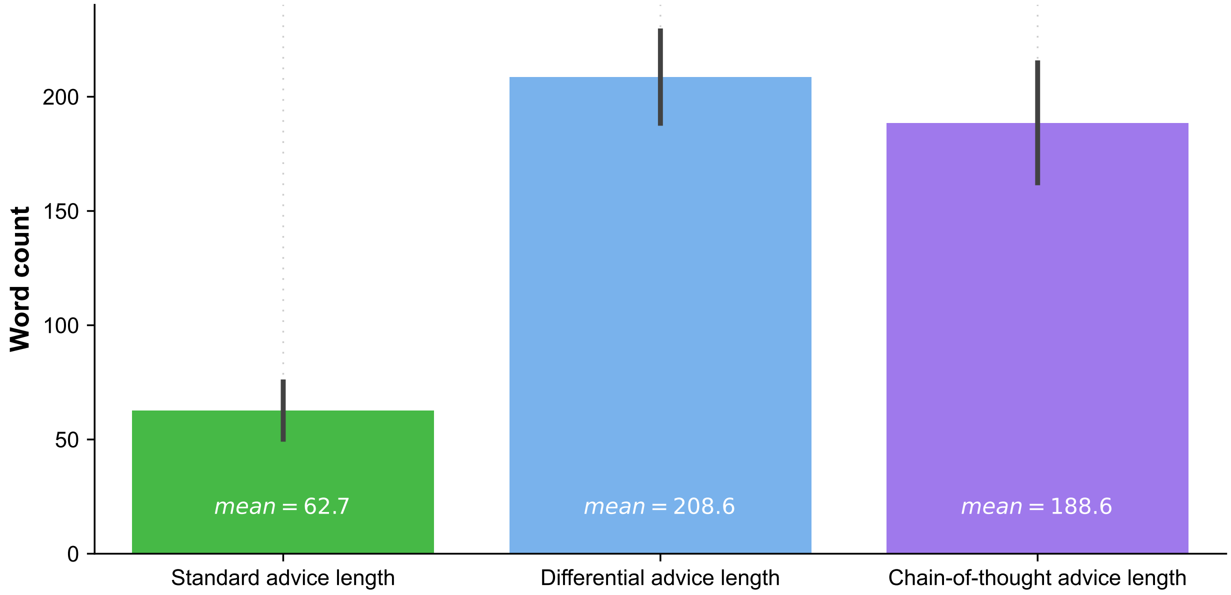

Supplementary Figure 2: **Length of LLM-generated explanations across different treatment groups (in words).** Bar plots show the mean word count across all patient cases (mean as text annotations). Whiskers denote the standard deviation. For each format, we generated 20 explanations corresponding to the $n = 20$ cases.

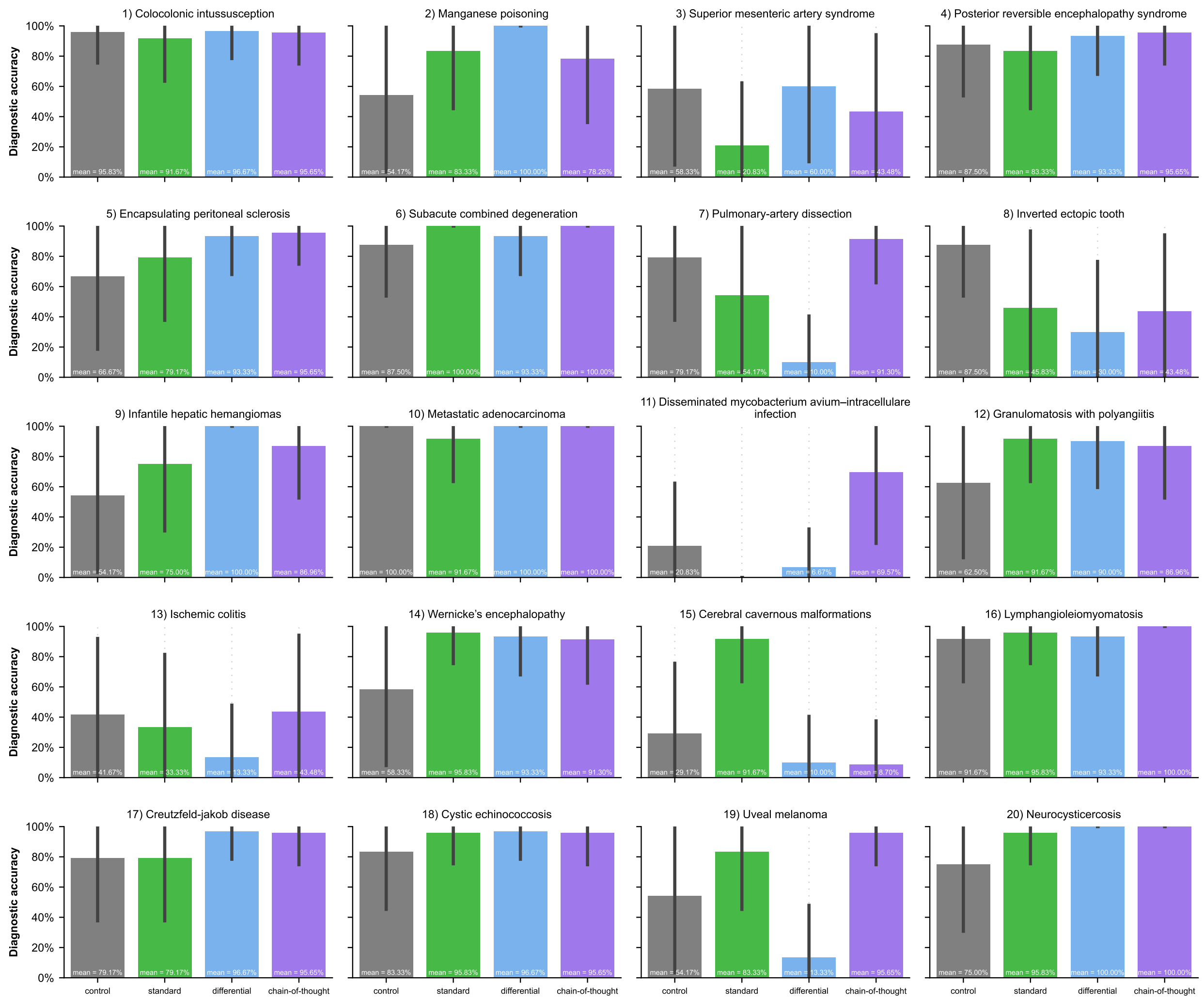

Supplementary Figure 3: **Diagnostic accuracy across patient cases.** For certain cases, such as *collagenous enterocolitis* and *superior mesenteric artery syndrome*, all conditions led to high diagnostic accuracy rates above 80%. However, other patient cases proved more challenging, with notably lower performance across all conditions. For instance, for the cases of *ischemic colitis* and *cerebral cavernous malformations*, diagnostic accuracy rates dropped below 40% for several conditions. The chain-of-thought group showed particularly strong performance in complex cases like *drug-induced thrombocytopenia* and *disseminated mycobacterium avium-intracellulare infection*, where other conditions often led to incorrect diagnoses. Physicians supported by the standard output performed well only in cases such as *Wernicke's encephalopathy* and *cystic echinococcosis*, while the differential diagnostic explanation showed strengths in diagnosing *pulmonary-artery dissection* and *infantile hepatic hemangioma*. This variation in performance across different medical conditions highlights the complexity of medical diagnosis and suggests that different prompting strategies might be more effective for different types of cases. Whiskers refer to standard deviations. For each patient case, the sample size by conditions is as follows: $n = 24$ for the control group, $n = 24$ for the standard condition, $n = 30$ for the differential diagnosis conditions, and $n = 23$ for the chain-of-thought condition.

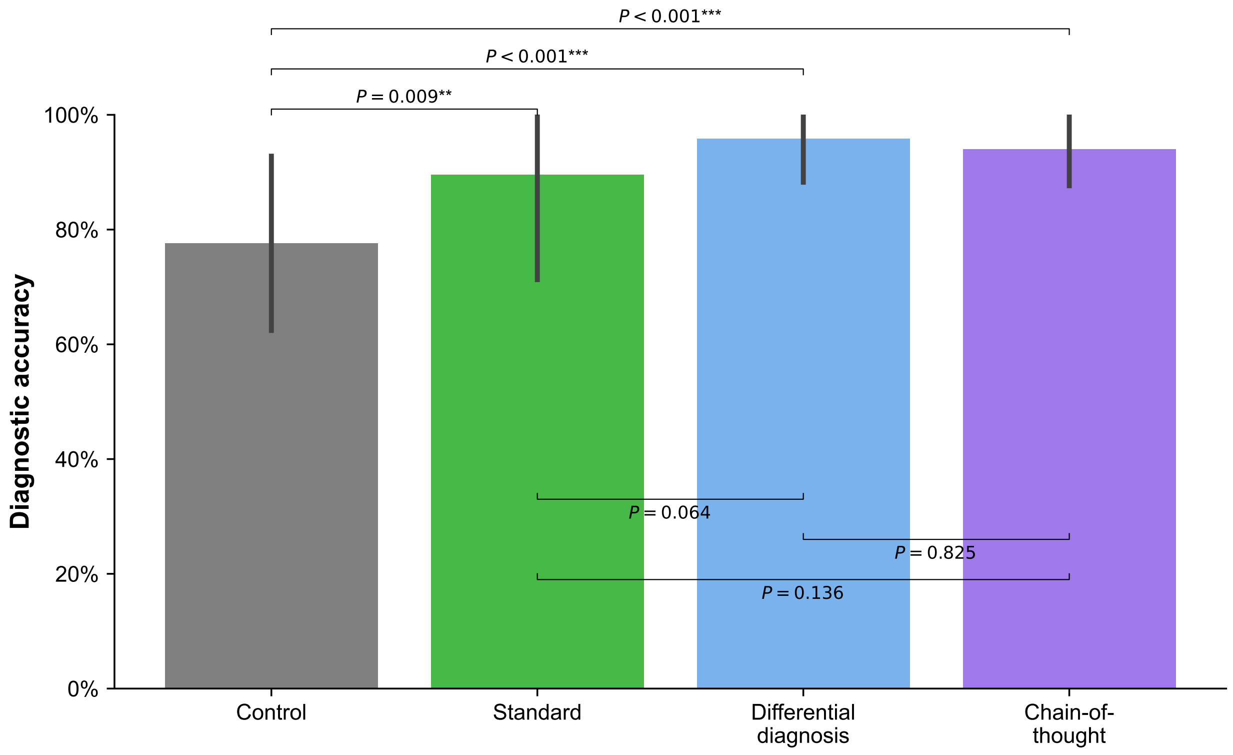

Supplementary Figure 4: **Diagnostic accuracy is shown for a subset of patient cases where LLM advice was uniformly correct (or incorrect) across all conditions.** Here, we restricted our analysis to the subset of patient cases where the LLM advice was correct (or incorrect) across all conditions. The figure shows the distribution of diagnostic accuracy for all participants as bar plots (which illustrate the probability density of the data). Whiskers show standard deviations. For the differential diagnosis condition, the top-5 diagnostic accuracy is reported. Statistical significance was assessed using one-sided Welch's t-test to compare diagnostic accuracy between the treatment groups (in the main text, we report results from a regression analysis to obtain effect sizes). The sample size is $n = 101$ aggregated at the participant level and, hence, does not contain repeated measures ($n = 24$ for the control group, $n = 24$ for the standard condition, $n = 30$ for the differential diagnosis conditions, and $n = 23$ for the chain-of-thought condition), however each observation is now the average diagnostic accuracy based on 8 patient case, instead of 20 cases.

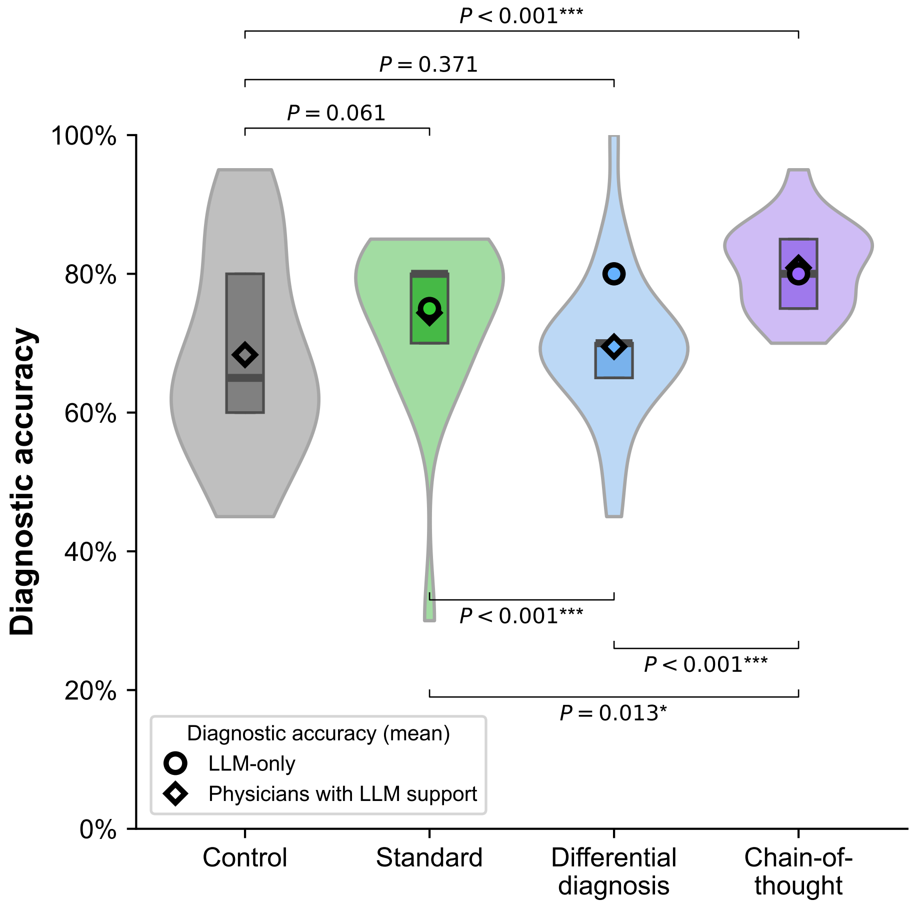

Supplementary Figure 5: **Robustness check for the diagnostic accuracy when counting partial responses as correct.** To account for diagnostic ambiguity (for instance, when a diagnosis appears accurate but omits a sub-diagnosis necessary for determining an appropriate treatment strategy), we perform a robustness check. The coding scheme in the main paper classified such partial responses as incorrect, while we now present an analysis in which we classified such responses as correct. The figure shows the distribution of diagnostic accuracy for all participants as violin plots (which illustrate the probability density of the data). The boxplots within each violin indicate the 25% and 75% quartiles, with the median represented by the thick center line. For the differential diagnosis condition, the top-5 diagnostic accuracy is reported (the top-1 diagnostic accuracy is lower and numbers to 65%). Statistical significance was assessed using one-sided Welch's t-test to compare diagnostic accuracy between the treatment groups. The sample size is $n = 101$ aggregated at the participant level and, hence, does not contain repeated measures ($n = 24$ for the control group, $n = 24$ for the standard condition, $n = 30$ for the differential diagnosis conditions, and $n = 23$ for the chain-of-thought condition).

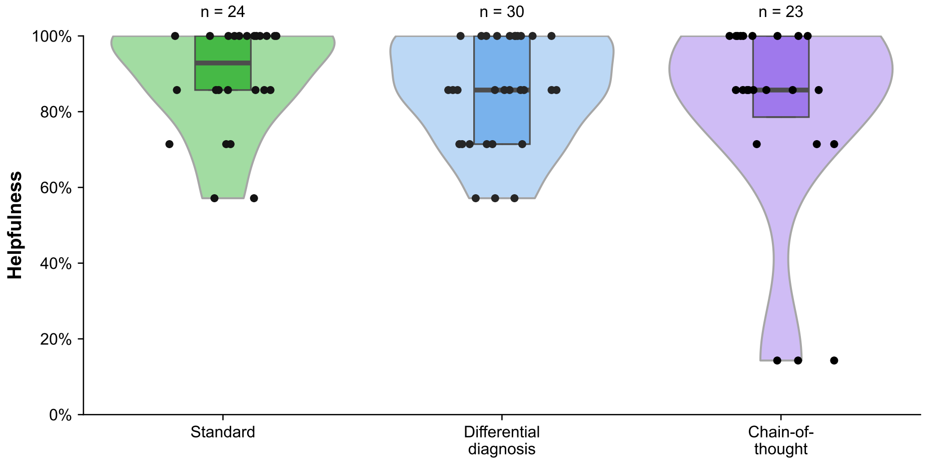

Supplementary Figure 6: **Effect on helpfulness.** The survey items were combined by averaging the Likert-scale ratings per participant and normalizing the result to a percentage scale. Helpfulness was not collected for the control group, leading to $n = 77$. Helpfulness was assessed by participants after completing the diagnostic tasks. An ANOVA reveals no statistically significant differences between the conditions (F-value = 1.324, P-value = 0.271), indicating that the type of explanation had no effect on helpfulness. Statistical significance was assessed using one-sided Welch's t-tests, showing no significant differences between any of the conditions (all P-values > 0.05). Whiskers denote standard deviations, there are no repeated measures.

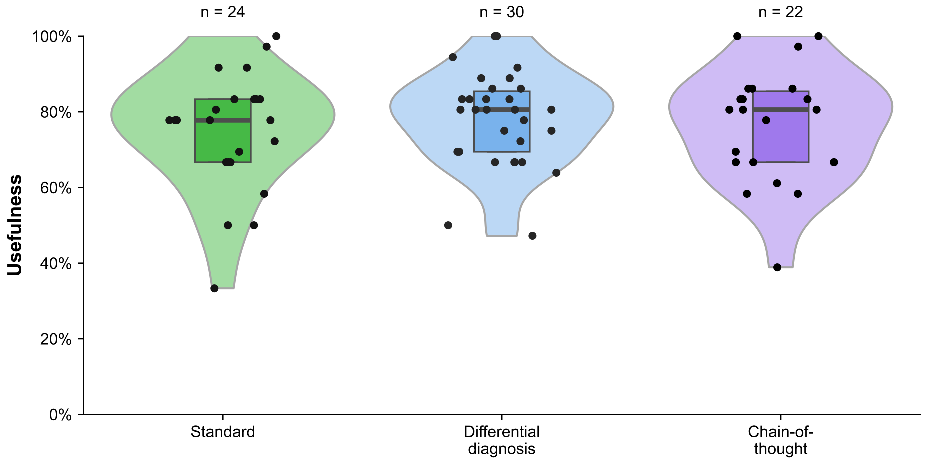

Supplementary Figure 7: **Effect on usefulness.** The survey items were combined by averaging the Likert-scale ratings per participant and normalizing the result to a percentage scale. The sample size of the post-study questionnaire varies, as the questionnaire was not fully displayed in two cases where participants selected specific specialties ("neuroradiologist" and "interventional radiology"). Usefulness was not collected for the control group, leading to $n = 76$. An ANOVA analysis reveals no statistically significant differences between the conditions (F-value = 0.690, P-value = 0.505), indicating that the explanation type had no effect on usefulness. Statistical significance was assessed using one-sided Welch's t-tests, showing no significant differences between any of the conditions (all P-values > 0.05). Whiskers denote standard deviations, there are no repeated measures.

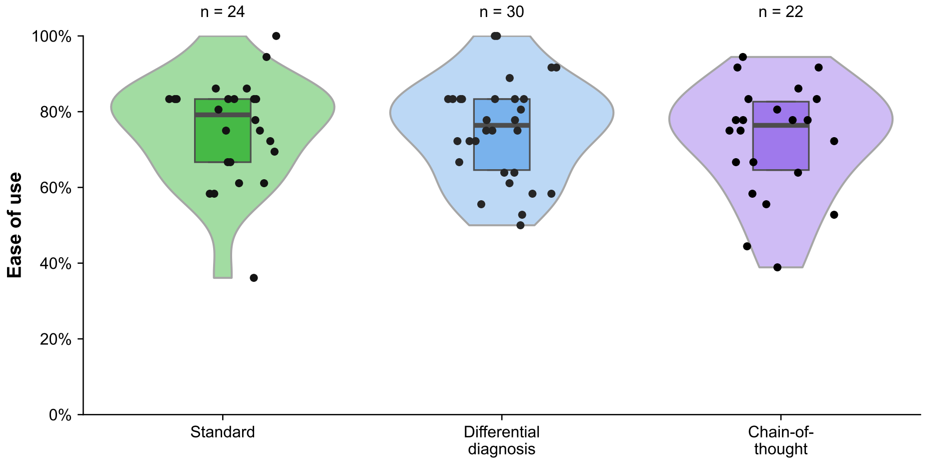

Supplementary Figure 8: **Effect on ease of use.** The survey items were combined by averaging the Likert-scale ratings per participant and normalizing the result to a percentage scale. The sample size of the post-study questionnaire varies, as the questionnaire was not fully displayed in two cases where participants selected specific specialties ("neuroradiologist" and "interventional radiology"). Ease of use was not collected for the control group, leading to $n = 76$. An ANOVA analysis reveals no statistically significant differences between the conditions (F-value = 1.313, P-value = 0.275), indicating that the explanation type had no effect on ease of use. Statistical significance was assessed using one-sided Welch's t-tests, showing no significant differences between any of the conditions (all P-values > 0.05). Whiskers denote standard deviations, there are no repeated measures.

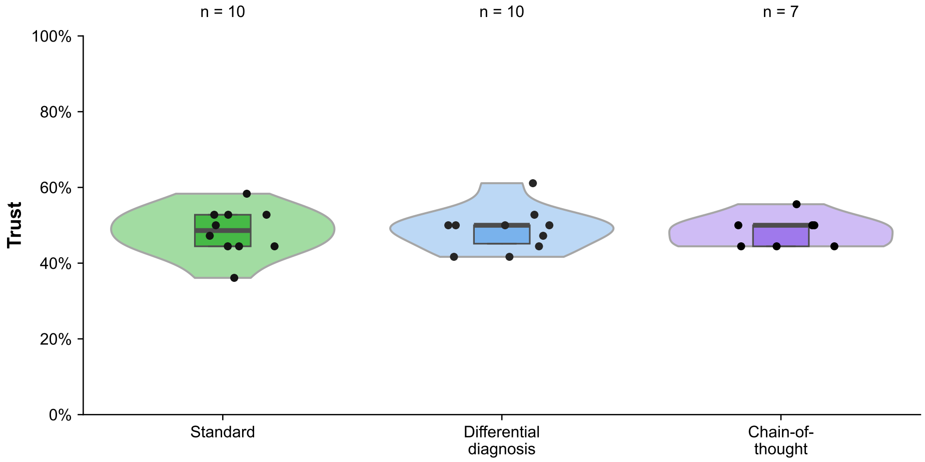

Supplementary Figure 9: **Effect on trust.** The survey items were combined by averaging the Likert-scale ratings per participant and normalizing the result to a percentage scale. The sample size for the trust construct is smaller due to a technical error in the study's setup, resulting in missing data. An ANOVA reveals no statistically significant differences between the conditions (F-value = 0.333, P-value = 0.718), indicating that the explanation type had no effect on trust. Statistical significance was assessed using one-sided Welch's t-tests, showing no significant differences between any of the conditions (all P-values > 0.05). Whiskers denote standard deviations, there are no repeated measures.

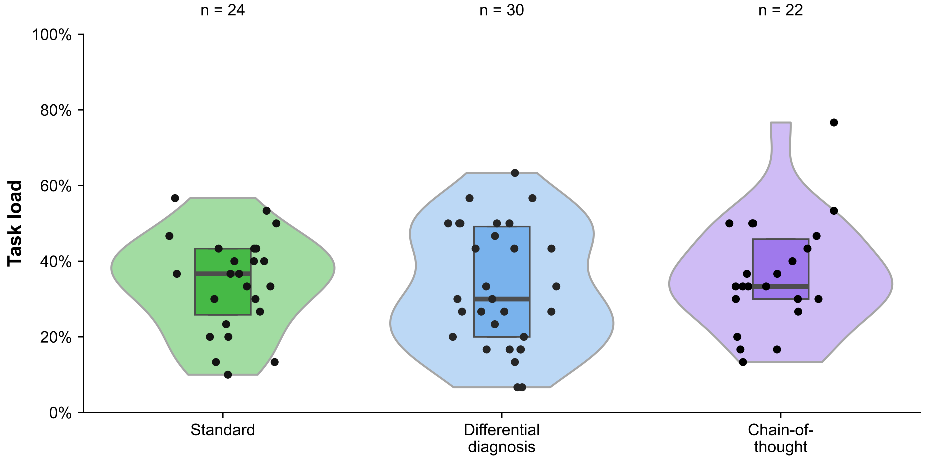

Supplementary Figure 10: **Effect on task load.** The survey items were combined by averaging the Likert-scale ratings per participant and normalizing the result to a percentage scale. The sample size of the post-study questionnaire varies, as the questionnaire was not fully displayed in two cases where participants selected specific specialties ("neuroradiologist" and "interventional radiology"), leading to $n = 99$. An ANOVA reveals no statistically significant differences between the conditions (F-value = 0.505, P-value = 0.679), indicating that the type of explanation had no effect on task load. Statistical significance was assessed using one-sided Welch's t-tests, showing no significant differences between any of the conditions (all P-values > 0.05). Whiskers denote standard deviations, there are no repeated measures.

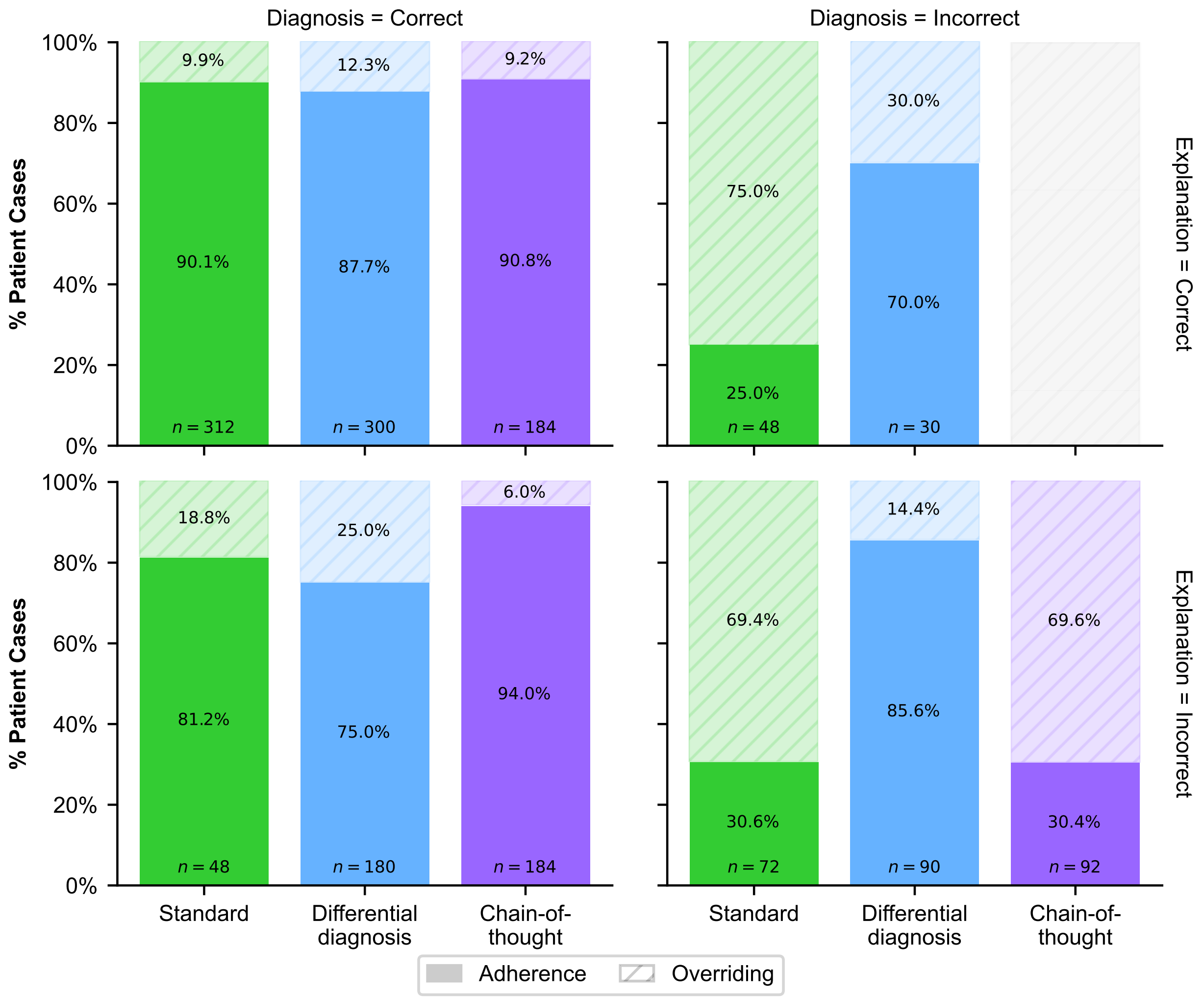

Supplementary Figure 11: **Adherence vs. overriding LLM advice for different explanation formats.** The figure depicts the breakdown of physician adherence (solid fill) and overriding (pattern fill) behaviors relative to LLM-generated diagnostic recommendations. Adherence was defined as the proportion of patient cases in which physicians' diagnoses followed the LLM-generated diagnoses, with overriding calculated as $1 - adherence$. Panels illustrate adherence by the correctness of the recommended diagnosis (columns) and correctness of the explanation provided by the LLM (rows), across the three conditions (standard, differential diagnosis, and chain-of-thought). Notably, diagnostic correctness of the LLM strongly influences adherence rates, highlighting increased over-adherence when diagnoses were incorrect, which is especially evident in the differential diagnosis condition. Conversely, the correctness of explanations shows only little impact on adherence patterns, suggesting that inaccuracies (hallucinations) in diagnoses contribute more significantly to inappropriate adherence than inaccuracies in explanations. The *correct explanation and incorrect diagnosis* scenario is absent as no LLM-generated advice met these criteria within the chain-of-thought group, which can be expected due to that chain-of-thought is designed to amend a given diagnosis with an explanation.

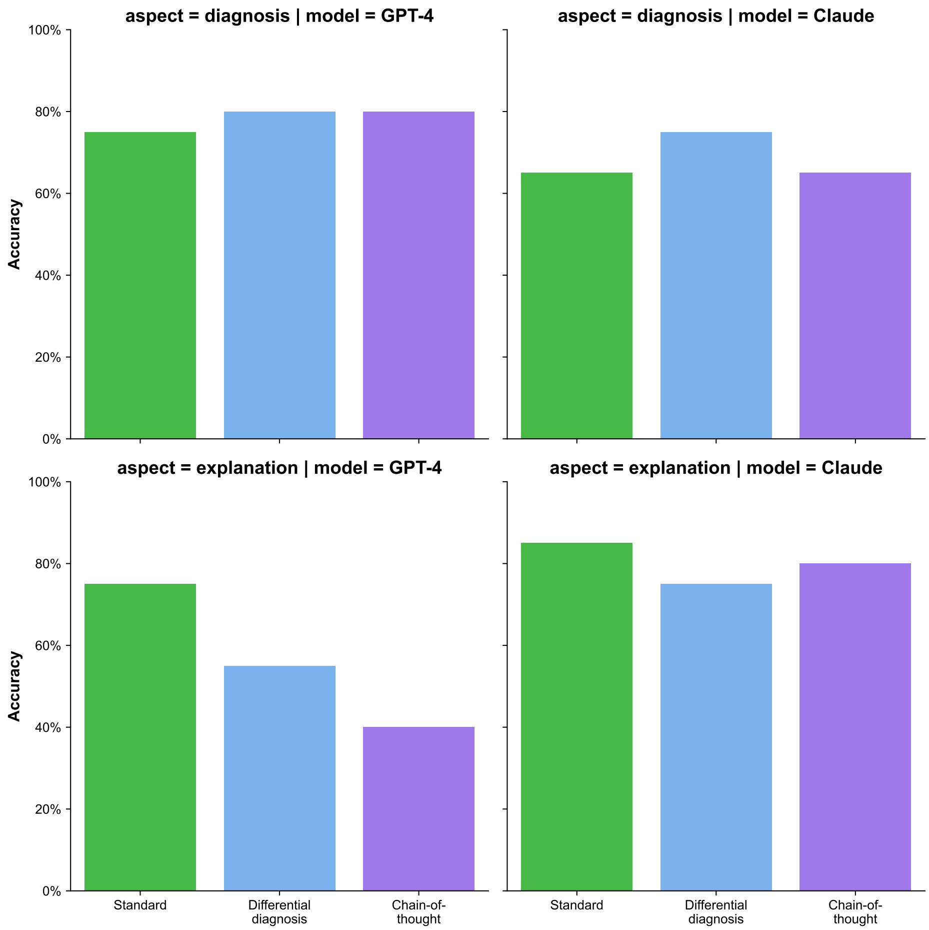

Supplementary Figure 12: **Comparison of GPT-4 and Claude in diagnosing patients.** To provide a justification for our choice of GPT-4, we now compare the diagnostic accuracy of GPT-4 against another state-of-the-art LLM, namely, Claude. The reason for the choice is that Claude is designed to handle multi-modal input (i.e., patient descriptions with radiology images). This is unlike many other open-source LLMs (e.g., Llama-3, DeepSeek-R1), which, to this date, are limited to text-only input. We generated advice for the 20 patient cases from our study with images and text using Claude (version claude-3-opus-20240229, temperature $= 1.0$, maximum token generations $= 4096$). We then assessed the accuracy of both the diagnoses and the explanations provided by the model. Overall, both GPT-4 and Claude are comparable in terms of diagnostic accuracy. The largest difference is observed in the accuracy of explanations. Considering that the effect of incorrect explanations on the participants in our study was small, it is reasonable to assume that we would obtain similar results from our study using a different LLM. To further support this claim, we calculated the inter-rater reliability of the models in diagnosing patient cases. The analysis of the inter-rater agreement between GPT-4 and Claude using Cohen's kappa revealed a fair agreement on both diagnoses ($\kappa = 0.243$) and explanations ($\kappa = 0.348$). The sample size for each condition on each subplot is $n = 20$, there are no repeated measures.

Supplementary Tables

Supplementary Table 1: **Prompts for generating explanations.** Shown are the three different prompting approaches for generating medical diagnosis explanations: standard output, differential diagnosis, and chain-of-thought reasoning

**Condition**	**Prompt**
Standard output	`Based on the provided text and image, I want you to make a diagnosis and explain your decision in 2-3 sentences.`
Differential diagnosis	`Differential diagnosis is a systematic method used by medical professionals to identify the underlying cause of a patient's symptoms or conditions and to eventually come up with a diagnosis based on a patient description and an image. It involves considering various possible diagnoses that could explain the symptoms and then systematically narrowing down the list based on clinical findings, medical history, laboratory tests, imaging studies, and other relevant information. The process typically involves the following steps: 1. Create a simple list with five possible diseases. 2. Loop through the list and explain why a specific disease does not fit, starting with the best fit and ending up with the disease that has the most unlikely fit. Reason for each disease within two short sentences taking into account the patient's symptoms. I want you to apply the process of differential diagnosis by taking into account not only the textual information but also the image.`
Chain-of-thought	`I want you to diagnose a patient by providing a diagnosis and explain how you come to that diagnosis step by step. For that, there is a patient description and an image with relevant information about the patient.`

Supplementary Table 2: **Items in the post-task survey.** Survey items measure participants' socio-demographics, expertise, and perceptions toward the LLM advice in the medical diagnosis tasks.

Variable	Items	Scale
Explanation helpfulness	• How helpful were the explanations?<br>• How can we make the AI advice more helpful for diagnosing patients?	7-point Likert-scale and free-form text answer respectively
Expertise in Generative AI	• How do you rate your expertise in using Generative AI like ChatGPT?	7-point Likert-scale
Specialization	• What is your specialization in medicine?<br>• Which area do you specialize in?	Multiple-choice-question with option for free-form text answer
Medical experience	• How many years of experience do you have as a medical professional?	Number
Experience in radiology	• How do you rate your experience in radiology?	7-point Likert-scale
IT skills	• How strong would you consider your IT skills?	7-point Likert-scale
Experience with medical AI systems	• How much experience do you have in working with medical AI systems?	7-point Likert-scale
Weekly visual inspection time	• How many hours per week do you spend on visual inspections?	7-point Likert-scale
Perceived self-efficacy	• Please estimate the percentage (0–100%) of diagnoses that you made correctly.<br>• How successful were you in accomplishing what you were asked to do?	Free-form text answer with check for numeric inputs and 7-point Likert-scale respectively
Perceived AI efficacy	• Please estimate the percentage (0–100%) of diagnoses that the AI system made correctly.<br>• How likely is the AI system to make a bad estimate?	Free-form text answer with check for numeric inputs and 7-point Likert-scale respectively
AI expectations	• How well did the AI system perform in comparison to your expectations?	7-point Likert-scale

Usefulness	• Using the AI system would enable others to classify architectural styles of buildings more quickly.<br>• Using the AI system would improve the performance when classifying architectural styles of buildings.<br>• Using the AI system would increase productivity for classifying the architectural styles of buildings.<br>• Using the AI system would enhance my effectiveness in classifying the architectural styles of buildings.<br>• Using the AI system would make it easier to classify the architectural styles of buildings.<br>• I would find the AI system useful for classifying the architectural styles of buildings.	7-point Likert-scale
Ease of use	• Learning to operate the AI would be easy for me.<br>• I would find it easy to get the AI to do what I want it to do.<br>• My interaction with the AI would be clear and understandable.<br>• I would find the AI to be flexible to interact with.<br>• It would be easy for me to become skillful at using AI.<br>• I would find the AI easy to use.	7-point Likert-scale
Trust	• The AI is deceptive.<br>• I am suspicious of the AI's intent, action, or outputs.<br>• The AI's actions will have a harmful outcome.<br>• I am confident in the AI.<br>• The AI is reliable.<br>• I can trust the AI.	7-point Likert-scale

Task load	• How mentally demanding was the task?<br>• How physically demanding was the task?<br>• How hurried or rushed was the pace of the task?<br>• How hard did you have to work to accomplish your level of performance?<br>• How insecure, discouraged, irritated, stressed, and annoyed were you?	7-point Likert-scale
Open-ended questions	• Have you used any assistive technology/tools (internet research, books, colleagues, etc.) in filling out this survey?<br>• In case you clicked "yes", which assistive technology/tools did you use?	Free-form text answer

Supplementary Table 3: **Patient cases included in the study.** Description of the 20 diverse patient cases used in the study, which include, for example, pediatric and geriatric patients with various presenting symptoms and diagnostic images.

Case 1	A previously healthy 5-year-old boy was brought to the surgery clinic with a 2-day history of intermittent abdominal pain. On palpation of the abdomen there was pain in the periumbilical region, but no rebound or guarding. An ultrasound was normal, and a computed tomography of the abdomen was performed (Panels A,B).
Case 2	A 55-year-old man presented with 10 years of progressive handwriting impairment and rapid, slurred speech. In his thirties, he had worked as a welder without access to personal protective equipment. Neurologic examination was notable for reduced facial expression, blepharospasm, and cluttered, dysarthric speech. Postural reflexes were mildly impaired. MRI imaging of the head showed a nonenhancing, T1-weighted, hyperintense signal in the basal ganglia on both sides. Ceruloplasmin and iron levels were normal.
Case 3	26-year-old man from Somalia presented with a 5-month history of dry cough, night sweats, and unintentional weight loss of 18 kg. During this period, epigastric pain and postprandial vomiting had also developed. His BMI was 11. On examination, he was cachectic with abdominal distention and diffuse tenderness to palpation. On the basis of chest imaging and sputum studies, a diagnosis of pulmonary tuberculosis was made, and intravenous antituberculous treatment was initiated. However, he continued to have postprandial vomiting. Contrast-enhanced CT of the abdomen was obtained.
Case 4	A 35-year-old man with IgA nephropathy presented with confusion, blurry vision, and seizures. Two weeks before presentation, he had started receiving cyclosporine. Physical examination was notable for a blood pressure of 160/80 mm Hg, drowsiness, and decreased visual acuity. A fundoscopic examinations was normal. T2-weighted magnetic resonance imaging (MRI) with fluid-attenuated inversion recovery sequencing of the head was performed.
Case 5	A 52-year-old woman with end-stage kidney disease that was being managed with peritoneal dialysis presented with a 1-month history of bloody dialysate. She had had 3 episodes of bacterial peritonitis in the past 12 years. Physical examination and laboratory studies were unremarkable. Computed tomography of the abdomen was performed.
Case 6	A 32-year-old man presented with a 6-week history of tingling in his arms and legs and a 2-week history of inability to walk. A positive Romberg test, sensory ataxia, impaired proprioception and vibratory sensation, and preserved nociception were noted. Magnetic resonance imaging of the whole spine showed hyperintensity in the posterior spinal cord from C1 to T12 and hyperintense lesions in the dorsal column on T2-weighted images. A vitamin B12 level was 107 pg per ml (reference value, >231) without macrocytic anemia.
Case 7	A 35-year-old woman with idiopathic pulmonary arterial hypertension and a pulmonary aneurysm presented with chest pain. Computed tomography (CT) of the chest is shown.
Case 8	A 38-year-old man presented to the otolaryngology clinic with chronic difficulty breathing through his right nostril. Physical examination showed nasal septal deviation, calcified septal spurs, and a 2-cm perforation in the posterior septum. On rhinoscopy, a hard, nontender, white mass was observed in the floor of the right nostril. CT of the paranasal sinuses showed a well-defined, radiodense mass.
Case 9	A 16-day-old girl was brought to the emergency department with lethargy. Physical exam showed tachypnea and marked hepatomegaly, as well as small hemangiomas on the skin. TSH was elevated. MRI showed numerous hepatic lesions and cardiomegaly.
Case 10	A 71-year-old man was hospitalized with altered mental status progressing over the preceding 3 weeks. The patient had a recent diagnosis of adenocarcinoma of the colon with known metastatic lesions in the lung and bones. A gadolinium-enhanced magnetic resonance image of the brain was performed and is shown.

Case 11	A 29-year-old man with perinatally acquired human immunodeficiency virus (HIV) infection and intermittent adherence to antiretroviral therapy presented to the hospital with abdominal pain and drenching night sweats. On presentation, his CD4 count was 18 cells per cubic millimeter (reference range, 500 to 1500), and the HIV viral load was undetectable. Physical exam showed severe abdominal distention, splenomegaly, and diffuse abdominal tenderness to palpation. Computed tomography of the abdomen confirmed massive splenomegaly with multifocal infarction of the splenic parenchyma.
Case 12	A 42-year-old man presented to the clinic with a 3-month history of worsening cough, shortness of breath, and fever. Physical examination showed inflamed nasal mucosa and nasal crusting. Wheezes and rales were heard on auscultation. A computed tomographic scan of the face showed extensive destruction of the structural bones of the midface, resulting in a large nasal cavity.
Case 13	A 63-year-old man presented to the emergency department with a 3-day history of abdominal pain that had started in the periumbilical area and subsequently shifted to the left lower quadrant. Initial laboratory tests showed a white-cell count of 12,000 per cubic millimeter (reference range, 4000 to 10,000) and a lactate level of 1.8 mmol per liter (normal value, <1.9). Contrast-enhanced computed tomography of the abdomen revealed edema of the sigmoid colon with thumbprinting.
Case 14	A 28-year-old woman with vertigo, confusion, and falls 2 weeks after a surgical abortion at 11 weeks of gestation presents to the emergency department. Examination revealed spontaneous upbeat nystagmus, gaze-evoked nystagmus, and gait ataxia.
Case 15	A 59-year-old previously healthy man presented with progressively worsening headaches and bluish nodular skin lesions. Fast-field echo MRI image of the brain showed this finding.
Case 16	A 44-year-old woman presented to the emergency department with acute chest pain after several months of progressive dyspnea. Her oxygen saturation was 92%, and she had diminished breath sounds on the right side. Chest CT revealed a large right-sided pneumothorax and diffuse, intraparenchymal pulmonary cysts.
Case 17	A 54-year-old man presented with a 3-week history of cognitive deterioration. Neurologic examination revealed disorientation, horizontal gaze-evoked nystagmus, hyperreflexia, startle myoclonus, and ataxia. Brain MRI with diffusion-weighted imaging revealed hyperintensity of the cortical gyri and caudate heads.
Case 18	A 30-year-old man presented with a 15-month history of intermittent discomfort in the right upper quadrant of the abdomen. He lived in a rural area of Morocco and had occasional contact with dogs. The physical examination revealed hepatomegaly with a palpable hepatic mass. Laboratory tests showed a normal white-cell count and a normal absolute eosinophil count. Ultrasonography and computed tomography of the abdomen revealed a large cyst in the right lobe of the liver.
Case 19	A 59-year-old woman presented to the emergency department with a 4-day history of inflammation and pain in the right eye. She had been blind in the eye for several years before presentation. Magnetic resonance imaging revealed a right orbital mass. Abdominal and thoracic imaging showed numerous hepatic masses, abdominal and thoracic lymphadenopathy, and vertebral sclerotic osseous disease. The right eye was enucleated for palliative relief and to obtain tissue for diagnosis.
Case 20	An 18-year-old man presented to the emergency department with generalized tonic–clonic seizures. On physical examination, the patient was confused. He had swelling over the right eye and tenderness in the right testis. Magnetic resonance imaging of the head showed numerous well-defined cystic lesions throughout the cerebral cortex.

Subspecialization	Frequency
General radiology	57
Abdominal imaging	24
Interventional radiology	20
Neuroradiology	20
Emergency radiology	19
Musculoskeletal radiology	18
Mammography / women's imaging	17
Ultrasound	15
Nuclear imaging	11
Head and neck radiology	9
Cardiothoracic imaging	8
Pediatric radiology	5
Other	4

Supplementary Table 4: **Number of radiologists by subspecialization.** Subspecialization was collected as a multi-option response format, meaning that participants could select multiple subspecializations. As a result, the total sample size in the above table does not equal the $n = 101$ recruited radiologists.

Subspecialization	Case	Diagnosis	General radiology knowledge
Abdominal imaging	Case 11	Disseminated Mycobacterium avium–intracellulare infection	✓
Abdominal imaging	Case 13	Ischemic colitis	✓
Abdominal imaging	Case 18	Cystic echinococcosis	✓
Abdominal imaging	Case 3	Superior mesenteric artery syndrome	✓
Abdominal imaging	Case 5	Encapsulating peritoneal sclerosis	
Cardiothoracic imaging	Case 16	Lymphangioleiomyomatosis	✓
Cardiothoracic imaging	Case 7	Pulmonary-artery dissection	
Head and neck radiology	Case 12	Granulomatosis with polyangiitis	✓
Head and neck radiology	Case 19	Uveal melanoma	✓
Head and neck radiology	Case 8	inverted ectopic tooth	
Neuroradiology	Case 10	Metastatic adenocarcinoma	✓
Neuroradiology	Case 14	Wernicke's encephalopathy	✓
Neuroradiology	Case 15	Cerebral cavernous malformations	✓
Neuroradiology	Case 17	Creutzfeld-Jakob disease	✓
Neuroradiology	Case 2	manganese poisoning	
Neuroradiology	Case 20	Neurocysticercosis	✓
Neuroradiology	Case 4	posterior reversible encephalopathy syndrome	✓
Neuroradiology	Case 6	subacute combined degeneration	✓
Pediatric radiology	Case 1	Colocolonic intussusception	✓
Pediatric radiology	Case 9	Infantile hepatic hemangiomas	✓

Supplementary Table 5: **Mapping of patient cases to subspecialization.** The specialty name is listed in the first column, followed by the corresponding question and diagnosis. The final column indicates whether the diagnosis is answerable based on general radiology education knowledge, assessed by our panel of radiologists based on common educational resources for radiologist training [1].

	Coef.	s.e.	P-value	95% CI
Intercept	1.386	0.559	0.013	[0.291; 2.482]
Condition: Standard	−0.288	0.761	0.705	[−1.779; 1.204]
Condition: Differential diagnosis	0.000	0.791	1.000	[−1.549; 1.549]
AIC				68.526
Obs. (N)				60

Supplementary Table 6: **Diagnostic accuracy of the LLM (without human involvement) across different prompting strategies.** We estimated a logistic regression at the diagnostic level (i.e., 20 diagnoses $\times$ 3 conditions). The chain of thought condition is used as the reference category. The odds ratios computed based on the coefficients are 0.750 for the standard condition and 1.000 for the differential diagnosis condition. Hence, considering the intercept, the chain-of-thought approach has a better diagnostic accuracy than the standard prompt and a similar diagnostic accuracy as the differential diagnosis. Abbreviations: s.e., standard error; CI, confidence interval, AIC, Akaike information criterion.

	Coef.	s.e.	P-value	95% CI
Intercept	0.617	0.024	< 0.001	[0.568; 0.665]
Condition: Standard	0.050	0.034	0.150	[−0.018; 0.118]
Condition: Differential diagnosis	0.025	0.033	0.446	[−0.040; 0.090]
Condition: Chain-of-thought	0.122	0.035	0.001	[0.053; 0.192]
AIC				−138.835
Obs. (N)				101

Supplementary Table 7: **Effect of explanation types on diagnostic accuracy.** OLS regression explaining the diagnostic accuracy at the physician level by the different conditions. The intercept represents the diagnostic accuracy of humans without LLM support (control condition). The sample size is $n = 101$ (i.e., diagnostic accuracy aggregated at the participant level; $n = 24$ for the control group, $n = 24$ for the standard output group, $n = 30$ for the differential diagnosis group, and $n = 23$ for the chain-of-thought group). Abbreviations: s.e., standard error; CI, confidence interval, AIC, Akaike information criterion.

	Coef.	s.e.	P-value	95% CI
Intercept	0.739	0.025	< 0.001	[0.690; 0.789]
Condition: Control	−0.122	0.035	0.001	[−0.192; −0.053]
Condition: Standard	−0.072	0.035	0.040	[−0.142; −0.003]
Condition: Differential diagnosis	−0.097	0.033	0.004	[−0.163; −0.032]
AIC				−138.835
Obs. (N)				101

Supplementary Table 8: **Benefit of chain-of-thought explanations on diagnostic accuracy.** OLS regression of diagnostic accuracy at the physician level on conditions with chain-of-thought as baseline (=intercept). This table re-parameterizes the model from Supplementary Supplementary Table 7 using chain-of-thought as the reference category to facilitate direct comparisons against the best-performing condition. The sample size is $n = 101$ (i.e., diagnostic accuracy aggregated at the participant level; $n = 24$ for the control group, $n = 24$ for the standard output group, $n = 30$ for the differential diagnosis group, and $n = 23$ for the chain-of-thought group). Abbreviations: s.e., standard error; CI, confidence interval, AIC, Akaike information criterion.

	Coef.	s.e.	P-value	95% CI
Intercept	0.604	0.123	< 0.001	[0.359; 0.850]
Condition: Standard	0.070	0.040	0.081	[−0.009; 0.149]
Condition: Differential diagnosis	0.039	0.039	0.325	[−0.039; 0.117]
Condition: Chain-of-thought	0.151	0.041	< 0.001	[0.070; 0.232]
Medical experience	0.001	0.002	0.707	[−0.003; 0.004]
Experience in radiology	−0.001	0.001	0.301	[−0.003; 0.001]
Experience with medical AI systems	−0.000	0.000	0.519	[−0.001; 0.001]
Weekly visual inspection time: 30−35h	0.077	0.108	0.478	[−0.138; 0.292]
Weekly visual inspection time: 35−40h	0.043	0.101	0.667	[−0.157; 0.244]
Weekly visual inspection time: 40−45h	0.067	0.100	0.508	[−0.133; 0.266]
Weekly visual inspection time: 45−50h	0.076	0.097	0.441	[−0.118; 0.269]
Weekly visual inspection time: 50−55h	0.039	0.100	0.698	[−0.159; 0.237]
Weekly visual inspection time: >55H	0.047	0.102	0.648	[−0.156; 0.249]
IT skills: Good	0.004	0.046	0.935	[−0.088; 0.095]
IT skills: Very Good	0.068	0.048	0.156	[−0.027; 0.163]
IT skills: Advanced	0.043	0.051	0.403	[−0.058; 0.143]
IT skills: Expert	0.023	0.072	0.748	[−0.120; 0.167]
AIC				−116.208
Obs. (N)				99

Supplementary Table 9: **Extended OLS regression of diagnostic accuracy with physician-level controls.** Reference category is the control group. In the post-survey questionnaire, the sample size of participants across conditions totals $n = 99$, as the questionnaire was not displayed in two cases where participants selected specific specialties ('neuroradiologist' and 'interventional radiology'). Hence, the two cases were dropped for the analysis. The sample size is $n = 99$ (i.e., diagnostic accuracy aggregated at the participant level; $n = 23$ for the control group, $n = 24$ for the standard output group, $n = 30$ for the differential diagnosis group, and $n = 22$ for the chain-of-thought group). In this analysis, we one-hot encoded responses for both weekly inspection time and IT skills; when treating these two variables as continuous, the results remain qualitatively the same, indicating that our results are robust to such changes. Abbreviations: s.e., standard error; CI, confidence interval, AIC, Akaike information criterion.

	Coef.	s.e.	P-value	95% CI
Intercept	0.544	0.041	< 0.001	[0.463; 0.625]
Condition: Standard output	0.021	0.028	0.467	[−0.035; 0.076]
Condition: Differential diagnosis	−0.048	0.017	0.005	[−0.081; −0.015]
Condition: Chain-of-thought	0.046	0.018	0.012	[0.010; 0.081]
Total time	0.000	0.000	0.125	[−0.000; 0.001]
Advice length	0.000	0.000	0.030	[0.000; 0.001]
Answer length	0.003	0.002	0.050	[−0.000; 0.006]
AIC				−141.197
Obs. (N)				101

Supplementary Table 10: **Extended OLS regression of diagnostic accuracy with advice-level controls.** Here, we control for total time (measured in minutes), advice length (measured in the number of words), and answer length (of the reported diagnosis by the physicians in the free-text form, measured in the number of characters). The reference category is the control group. The sample size is $n = 101$ (i.e., diagnostic accuracy aggregated at the participant level; $n = 24$ for the control group, $n = 24$ for the standard output group, $n = 30$ for the differential diagnosis group, and $n = 23$ for the chain-of-thought group). Abbreviations: s.e., standard error; CI, confidence interval, AIC, Akaike information criterion.

	Coef.	s.e.	P-value	95% CI
Intercept	0.575	0.037	< 0.001	[0.500; 0.650]
Condition: Standard	0.025	0.051	0.628	[-0.079; 0.129]
Condition: Differential diagnosis	0.090	0.052	0.094	[-0.016; 0.196]
Condition: Chain-of-thought	0.167	0.050	0.002	[0.065; 0.268]
AIC				-58.545
Obs. (N)				43

Supplementary Table 11: **Subgroup analysis for participants with basic IT skills.** OLS regression explaining the diagnostic accuracy at the physician level by the conditions for the subgroup of participants that have basic IT skills (i.e., lower or equal to "Good"). Reference category is the control group. The sample size is $n = 43$ (i.e., diagnostic accuracy aggregated at the participant level; $n = 10$ for the control group, $n = 11$ for the standard output group, $n = 10$ for the differential diagnosis group, and $n = 12$ for the chain-of-thought group). Abbreviations: s.e., standard error; CI, confidence interval, AIC, Akaike information criterion.

	Coef.	s.e.	P-value	95% CI
Intercept	0.646	0.033	< 0.001	[0.581; 0.711]
Condition: Standard	0.077	0.046	0.101	[−0.015; 0.169]
Condition: Differential diagnosis	−0.016	0.042	0.701	[−0.100; 0.068]
Condition: Chain-of-thought	0.094	0.049	0.063	[−0.005; 0.193]
AIC				−77.177
Obs. (N)				56

Supplementary Table 12: **Subgroup analysis for participants with advanced IT skills.** OLS regression explaining the diagnostic accuracy at the physician level by the conditions for the subgroup of participants that have advanced IT skills (i.e., better than "Good"). Reference category is the control group. The sample size is $n = 56$ (i.e., diagnostic accuracy aggregated at the participant level; $n = 24$ for the control group, $n = 24$ for the standard output group, $n = 30$ for the differential diagnosis group, and $n = 23$ for the chain-of-thought group). Abbreviations: s.e., standard error; CI, confidence interval, AIC, Akaike information criterion.

	Coef.	s.e.	P-value	95% CI
Intercept	0.619	0.030	< 0.001	[0.558; 0.680]
Condition: Standard	0.072	0.040	0.077	[−0.008; 0.153]
Condition: Differential diagnosis	0.037	0.036	0.318	[−0.037; 0.110]
Condition: Chain-of-thought	0.109	0.042	0.012	[0.025; 0.193]
AIC				−91.631
Obs. (N)				46

Supplementary Table 13: **Subgroup analysis for participants with short tenure.** OLS regression explaining the diagnostic accuracy at the physician level by the conditions for the subgroup of radiologists with short tenure (less than 12.0 years). Reference category is the control group. The sample size is $n = 46$ (i.e., diagnostic accuracy aggregated at the participant level; $n = 8$ for the control group, $n = 11$ for the standard output group, $n = 18$ for the differential diagnosis group, and $n = 9$ for the chain-of-thought group). Abbreviations: s.e., standard error; CI, confidence interval, AIC, Akaike information criterion.

	Coef.	s.e.	P-value	95% CI
Intercept	0.613	0.038	< 0.001	[0.537; 0.689]
Condition: Standard	0.033	0.055	0.556	[−0.078; 0.144]
Condition: Differential diagnosis	0.008	0.057	0.895	[−0.106; 0.121]
Condition: Chain-of-thought	0.137	0.055	0.017	[0.025; 0.248]
AIC				−49.587
Obs. (N)				53

Supplementary Table 14: **Subgroup analysis for participants with long tenure.** OLS regression explaining the diagnostic accuracy at the physician level by the conditions for the subgroup of radiologists with long tenure (more than or equal to 12.0 years). Reference category is the control group. The sample size is $n = 53$ (i.e., diagnostic accuracy aggregated at the participant level; $n = 15$ for the control group, $n = 13$ for the standard output group, $n = 12$ for the differential diagnosis group, and $n = 13$ for the chain-of-thought group). Abbreviations: s.e., standard error; CI, confidence interval, AIC, Akaike information criterion.

	Coef.	s.e.	P-value	95% CI
Intercept	0.867	0.022	< 0.001	[0.823; 0.911]
Condition: Standard	−0.033	0.031	0.291	[−0.096; 0.029]
Condition: Differential diagnosis	−0.057	0.030	0.060	[−0.116; 0.002]
Condition: Chain-of-thought	0.051	0.032	0.113	[−0.012; 0.114]
AIC				−157.675
Obs. (N)				101

Supplementary Table 15: **Subgroup analysis for basic patient cases.** OLS regression explaining the diagnostic accuracy at the physician level by the conditions for the subset of basic cases. We split patient cases into basic and complex subsets based on the mean diagnostic accuracy of the control group for each case, where the lower half corresponds to the basic cases. Reference category is the control group. The sample size is $n = 101$ (i.e., diagnostic accuracy aggregated at the participant level; $n = 24$ for the control group, $n = 24$ for the standard output group, $n = 30$ for the differential diagnosis group, and $n = 23$ for the chain-of-thought group). Abbreviations: s.e., standard error; CI, confidence interval, AIC, Akaike information criterion.

	Coef.	s.e.	P-value	95% CI
Intercept	0.500	0.031	< 0.001	[0.438; 0.562]
Condition: Standard	0.154	0.045	0.001	[0.066; 0.243]
Condition: Differential diagnosis	0.080	0.042	0.061	[−0.004; 0.164]
Condition: Chain-of-thought	0.200	0.045	< 0.001	[0.111; 0.289]
AIC				−87.045
Obs. (N)				101

Supplementary Table 16: **Subgroup analysis for complex patient cases.** OLS regression explaining the diagnostic accuracy at the physician level by the conditions for the subset of complex cases. We split patient cases into basic and complex subsets based on the mean diagnostic accuracy of the control group for each case, where the upper half corresponds to the complex cases. Reference category is the control group. The sample size is $n = 101$ (i.e., diagnostic accuracy aggregated at the participant level; $n = 24$ for the control group, $n = 24$ for the standard output group, $n = 30$ for the differential diagnosis group, and $n = 23$ for the chain-of-thought group). Each diagnostic accuracy is calculated based on the 10 complex patient cases. Abbreviations: s.e., standard error; CI, confidence interval, AIC, Akaike information criterion.

	Coef.	s.e.	P-value	95 % CI
Intercept	0.629	0.034	< 0.001	[0.562; 0.697]
Condition: Standard output	0.048	0.040	0.241	[−0.032; 0.128]
Condition: Differential diagnosis	0.032	0.038	0.398	[−0.043; 0.108]
Condition: Chain-of-thought	0.133	0.039	0.001	[0.056; 0.211]
Abdominal imaging	−0.015	0.039	0.699	[−0.092; 0.062]
Cardiothoracic imaging	0.038	0.058	0.516	[−0.077; 0.152]
Emergency radiology	0.004	0.037	0.917	[−0.070; 0.078]
General radiology	−0.004	0.027	0.891	[−0.058; 0.051]
Head and neck radiology	−0.022	0.063	0.733	[−0.147; 0.104]
Interventional radiology	−0.048	0.035	0.175	[−0.117; 0.022]
Mammography / women's imaging	−0.005	0.037	0.883	[−0.079; 0.068]
Musculoskeletal radiology	−0.044	0.037	0.236	[−0.118; 0.030]
Neuroradiology	0.020	0.043	0.636	[−0.065; 0.105]
Nuclear Imaging	−0.012	0.045	0.791	[−0.102; 0.078]
Pediatric Radiology	−0.072	0.061	0.243	[−0.194; 0.050]
Ultrasound	0.062	0.050	0.216	[−0.037; 0.161]
Other	−0.065	0.068	0.341	[−0.200; 0.070]
AIC				−120.762
Obs. (N)				101

Supplementary Table 17: **OLS regression controlling for radiologist subspecialization.** Here, we follow the OLS regression from the main paper (to estimate the effect compared to the control group) but additionally control for the subspecialization of the physicians (as defined in Supplementary Supplementary Table 5; subspecializations with no response are dropped from the table). None of the dummies controlling for subspecialization shows significant differences, implying that the differences in radiologists' backgrounds cannot explain away our main findings. Abbreviations: s.e., standard error; CI, confidence interval, AIC, Akaike information criterion.

	Coef.	s.e.	P-value	95 % CI
Intercept	0.696	0.030	< 0.001	[0.636; 0.756]
Standard output	0.079	0.041	0.056	[−0.002; 0.160]
Differential diagnosis	0.037	0.039	0.351	[−0.041; 0.115]
Chain-of-thought	0.130	0.043	0.003	[0.044; 0.216]
AIC				−99.749
Obs. (N)				77

Supplementary Table 18: **OLS regression explaining the explanation format effect on the diagnostic accuracy for patient cases within participants' subspecialties.** The analysis aims to analyze the scenario in which physicians only diagnose patients in their subspecialty (as defined in Supplementary Supplementary Table 5). For this, we run the regression (with the control condition as reference) on the relevant, subspecialty-matched subset of the data, which we obtained as follows. The 20 patient cases were assigned to the subspecialties as follows (see Supplementary Supplementary Table 5): 8 cases to neurology, 3 to head and neck radiology, 5 to abdominal imaging, 2 to cardiothoracic imaging, and 2 to pediatric radiology. Additionally, of the 20 cases, 16 are identified to be answerable based on general radiology knowledge obtained through widespread radiology education resources, such as *"Core Radiology: A Visual Approach to Diagnostic Imaging"* [1], and therefore included in the diagnostic accuracy assessment of all specialized participants. Participants without a focus on general radiology or at least one specialty covered by our patient cases are excluded in this analysis, resulting in a sample size of $n = 77$. We then assess the diagnostic accuracy of the participants based on cases within each participant's subspecialty. Abbreviations: s.e., standard error; CI, confidence interval; AIC, Akaike information criterion.

	Coef.	s.e.	P-value	95 % CI
Intercept	0.650	0.044	< 0.001	[0.562; 0.738]
Condition: Standard output	0.009	0.054	0.862	[−0.098; 0.117]
Condition: Differential diagnosis	−0.000	0.052	1.000	[−0.104; 0.104]
Condition: Chain-of-thought	0.077	0.056	0.172	[−0.034; 0.188]
AIC				−72.764
Obs. (N)				57

Supplementary Table 19: **OLS regression of explanation format effect on the diagnostic accuracy among general radiologists.** The model is estimated on $n = 57$ participants who self-identify as general radiologists. Abbreviations: s.e., standard error; CI, confidence interval; AIC, Akaike information criterion.

	Coef.	s.e.	P-value	95% CI
Intercept	0.625	0.021	< 0.001	[0.584; 0.666]
Condition: Standard	0.052	0.029	0.079	[−0.006; 0.110]
Condition: Differential diagnosis	0.015	0.028	0.591	[−0.040; 0.070]
Condition: Chain-of-thought	0.114	0.030	< 0.001	[0.055; 0.173]
AIC				−171.332
Obs. (N)				101

Supplementary Table 20: **Robustness checks controlling for outliers.** OLS regression explaining the winsorized diagnostic accuracy at the physician level by the different conditions. To improve the statistical reliability and reduce the potential bias from outliers or extreme values, we applied winsorization to the diagnostic accuracy at the participant level. To improve statistical reliability and reduce bias from extreme values, we winsorized each physician's diagnostic accuracy (i.e., proportion correct across 20 cases) by replacing values below the 5th percentile or above the 95th percentile with the values at these percentiles. Here, the reference category is the control group. The sample size is $n = 101$ (i.e., diagnostic accuracy aggregated at the participant level; $n = 24$ for the control group, $n = 24$ for the standard output group, $n = 30$ for the differential diagnosis group, and $n = 23$ for the chain-of-thought group). Abbreviations: s.e., standard error; CI, confidence interval, AIC, Akaike information criterion.

	Coef.	s.e.	P-value	95% CI
Intercept	1.036	0.823	0.208	[−0.577; 2.648]
Condition: Standard	0.548	0.248	0.027	[0.062; 1.034]
Condition: Differential diagnosis	0.095	0.242	0.695	[−0.379; 0.569]
Condition: Chain-of-thought	1.141	0.259	< 0.001	[0.633; 1.650]
Medical experience	0.001	0.011	0.922	[−0.021; 0.023]
Experience in radiology	−0.008	0.007	0.256	[−0.021; 0.006]
Experience with medical AI systems	−0.003	0.003	0.316	[−0.009; 0.003]
Weekly visual inspection time: 30−35h	0.738	0.663	0.265	[−0.561; 2.038]
Weekly visual inspection time: 35−40h	0.361	0.610	0.554	[−0.834; 1.556]
Weekly visual inspection time: 40−45h	0.551	0.607	0.364	[−0.639; 1.741]
Weekly visual inspection time: 45−50h	0.535	0.590	0.364	[−0.621; 1.691]
Weekly visual inspection time: 50−55h	0.299	0.604	0.621	[−0.884; 1.482]
Weekly visual inspection time: >55h	0.314	0.617	0.611	[−0.896; 1.524]
IT skills: Good	0.070	0.285	0.805	[−0.488; 0.628]
IT skills: Very Good	0.555	0.299	0.063	[−0.030; 1.140]
IT skills: Advanced	0.516	0.316	0.103	[−0.104; 1.136]
IT skills: Expert	0.330	0.452	0.465	[−0.556; 1.215]
AIC				1780.944
Obs. (N)				1980
Variance explained by participant IDs				0.235
Variance explained by case IDs				2.275

Supplementary Table 21: **Mixed-effects model.** We estimated a mixed-effects model to control for heterogeneity across physicians and cases. Reference category is the control group. As we control for variables assessed in the questionnaire, which have not been displayed in two cases, the sample size is reduced to $N = 1980 = 2020 - 20 \times 2$ assessments ($n = 460$ for the control group, $n = 480$ for the standard output group, $n = 600$ for the differential diagnosis group, and $n = 440$ for the chain-of-thought group), which 20 observations belonging to each participant. Abbreviations: s.e., standard error; CI, confidence interval, AIC, Akaike information criterion.

	Coef.	s.e.	P-value	95% CI
Intercept	0.475	0.105	< 0.001	[0.269; 0.681]
Condition: Standard output	0.218	0.151	0.149	[−0.078; 0.514]
Condition: Differential diagnosis	0.107	0.142	0.450	[−0.171; 0.385]
Condition: Chain-of-thought	0.566	0.159	< 0.001	[0.255; 0.877]
AIC				95.364
Obs. (N)				101

Supplementary Table 22: **Quasi-binomial regression.** We estimated quasi-binomial regression, which may limit the interpretability of the estimated effect sizes but which can better accommodate for the fact that the diagnostic accuracy is in the range between 0% and 100%. Here, we fitted a generalized linear model with a logistic link function, using a binomial error distribution to model the diagnostic accuracy of responses based on the conditions from our experiment. We further adjusted for overdispersion by scaling the standard errors using a dispersion factor estimated from the Pearson χ^2-statistic. Reference category is the control group. The sample size is $n = 101$ (i.e., diagnostic accuracy aggregated at the participant level; $n = 24$ for the control group, $n = 24$ for the standard output group, $n = 30$ for the differential diagnosis group, and $n = 23$ for the chain-of-thought group). Abbreviations: s.e., standard error; CI, confidence interval, AIC, Akaike information criterion.

	Coef.	s.e.	P-value	95% CI
Intercept	79.369	30.649	0.012	[18.327; 140.411]
Condition: Standard	13.117	43.344	0.763	[−73.209; 99.443]
Condition: Differential diagnosis	68.332	43.344	0.119	[−17.994; 154.659]
Condition: Chain-of-thought	57.825	43.344	0.186	[−28.501; 144.152]
AIC				1018.199
Obs. (N)				80

Supplementary Table 23: **Effect on decision time.** OLS regression explaining the average time to answer each case across the different conditions, leading to $N = 80$ observations (= 20 cases $\times$ 4 conditions). Reference category is the control group. Abbreviations: s.e., standard error; CI, confidence interval, AIC, Akaike information criterion.

	Coef.	s.e.	P-value	95 % CI
Intercept	92.485	34.900	0.010	[22.599; 162.372]
Condition: Differential diagnosis	55.216	49.356	0.268	[−43.619; 154.050]
Condition: Chain-of-thought	44.709	49.356	0.369	[−54.126; 143.543]
AIC				779.238
Obs. (N)				60

Supplementary Table 24: **Effect of decision time (compared to the standard group).** OLS regression explaining the average time to answer each case across the different conditions. The standard output group is set as the reference group, leading to $N = 60$ observations (= 20 cases $\times 3$ treatment groups). Abbreviations: s.e., standard error; CI, confidence interval, AIC, Akaike information criterion.

	Coef.	s.e.	P-value	95% CI
Intercept	0.769	0.098	< 0.001	[0.577; 0.961]
Condition: Standard	0.296	0.143	0.039	[0.015; 0.577]
Condition: Differential diagnosis	0.054	0.132	0.680	[-0.205; 0.314]
Condition: Chain-of-thought	0.672	0.154	< 0.001	[0.371; 0.974]
AIC				2340.802
Obs. (N)				2020

Supplementary Table 25: **Logistic regression at the assessment level.** Logistic regression at the assessment level (=1 if the correct diagnosis was given by a physician for a specific case, 0 otherwise) explaining the diagnostic accuracy by the different conditions. Reference category is the control group. The sample size consists of $N = 2020$ observations, each participant measured 20 times ($N = 480$ for the control group, $N = 480$ for the standard output group, $N = 600$ for the differential diagnosis group, and $N = 460$ for the chain-of-thought group). Abbreviations: s.e., standard error; CI, confidence interval, AIC, Akaike information criterion.

	Coef.	s.e.	P-value	95% CI
Intercept	−0.619	0.171	< 0.001	[−0.954; −0.285]
Condition: Standard	−0.504	0.209	0.016	[−0.914; −0.094]
Condition: Differential diagnosis	−1.014	0.188	< 0.001	[−1.383; −0.646]
Correct explanation ($True = 1$)*: 1*	0.462	0.162	0.004	[0.144; 0.779]
Correct diagnosis ($True = 1$)*: 1*	2.900	0.168	< 0.001	[2.571; 3.229]
AIC				1263.086
Obs. (N)				1540

Supplementary Table 26: **Logistic regression controlling for correct explanations and correct diagnosis in the LLM advice.** Logistic regression at the assessment level (=1 if the correct diagnosis was given by a physician for a specific case, 0 otherwise) explaining the diagnostic accuracy by the different conditions. Here, we separately control for (a) whether the suggested diagnosis of the LLM is correct or (b) whether the explanation generated by the LLM is correct. The diagnosis in the differential diagnosis group is considered correct if the correct diagnosis is one of the five presented options. The chain-of-thought group served as the reference group. We omit the control group because the control did not receive LLM advice, making it impossible to control for the correctness of that advice. The sample size consists of $N = 1540$ observations, for which 20 observations belong to each participant ($N = 480$ for the standard output group, $N = 600$ for the differential diagnosis group, and $N = 460$ for the chain-of-thought group). Abbreviations: s.e., standard error; CI, confidence interval, AIC, Akaike information criterion.

	Coef.	s.e.	P-value	95 % CI
Condition: Standard	0.470	0.137	0.001	[0.201; 0.739]
Condition: Differential diagnosis	0.216	0.125	0.085	[−0.030; 0.461]
Condition: Chain-of-thought	0.862	0.148	< 0.001	[0.572; 1.152]
Image: Count	0.045	0.075	0.546	[−0.102; 0.192]
Source: MRI	0.729	0.141	< 0.001	[0.451; 1.006]
Body region: Head	0.192	0.136	0.159	[−0.075; 0.460]
Body region: Spine	1.806	0.488	< 0.001	[0.849; 2.762]
Body region: Thorax	0.719	0.176	< 0.001	[0.375; 1.064]
AIC				2267.722
Obs. (N)				2020

Supplementary Table 27: **Regression controlling for the characteristics of radiological images.** Logistic regression at the assessment level (=1 if the correct diagnosis was given by a physician for a specific case, 0 otherwise) explaining the diagnostic accuracy by the different conditions and image characteristics. Here, we include (a) the number of images shown for a patient case, (b) the source of the image(s) (i.e., MRI or CT [CT serves as a baseline in the regression]), and (c) the body region shown in the image(s) (abdomen serves as a baseline for the regression here). By exponentiating the coefficients, the odds ratios of 1.60 for the standard condition, 1.24 for the differential condition, and 2.37 for the chain-of-thought condition are obtained. The sample size consists of $N = 2020$ observations, with 20 observations per participant ($N = 480$ for the control group, $N = 480$ for the standard group, $n = 600$ for the differential diagnosis group, and $n = 460$ for the chain-of-thought group). Abbreviations: s.e., standard error; CI, confidence interval.

Variable	Statistic	Control	Standard	Differential	Chain-of-thought	Overall
Medical Experience	Mean	15.087	13.104	10.800	16.500	13.621
	SD	6.557	6.617	7.364	10.327	7.981
Medical AI Experience	Mean	5.000	5.500	5.133	4.818	5.121
	SD	1.859	1.956	1.814	2.322	1.965
Radiology Experience	Mean	5.739	6.000	5.967	6.500	6.040
	SD	1.010	0.933	0.964	0.598	0.925
IT Skills	Top	Very Good	Good	Good	Good	Good
	Freq	10	8	9	9	33
Weekly Inspection Time	Top	45-50h	>55h	35-40h	45-50h	45-50h
	Freq	8	7	7	5	25

Supplementary Table 28: **Demographics of study sample by condition.** The table presents demographic statistics for study participants across four experimental conditions. Variables include medical experience (years, freely reported), medical AI experience (reported on a 7-point Likert-scale, normalized to values between 0 and 100), radiology experience (reported on a 7-point Likert-scale, normalized to values between 0 and 100), and weekly inspection time (reported on a 7-point Likert-scale, converted to the categories displayed in the study). In the table, we report the mean and standard deviation (SD) for continuous variables. For categorical variables, we report the most frequently occurring category (top) and its frequency (freq).

Supplementary References

[1] Sun, E. X., Shi, J. & Mandell, J. C. (eds.) *Core radiology: a visual approach to diagnostic imaging* (Cambridge University Press, 2021), 2 edn.
